# Clofazimine enhances the efficacy of BCG revaccination via stem cell-like memory T cells

**DOI:** 10.1371/journal.ppat.1008356

**Published:** 2020-05-21

**Authors:** Shaheer Ahmad, Debapriya Bhattacharya, Neeta Gupta, Varsha Rawat, Sultan Tousif, Luc Van Kaer, Gobardhan Das

**Affiliations:** 1 Special Centre for Molecular Medicine, Jawaharlal Nehru University, New Delhi, India; 2 Department of Pathology, Microbiology and Immunology, Vanderbilt University School of Medicine, Nashville, Tennessee, United States of America; Portland VA Medical Center, Oregon Health and Science University, UNITED STATES

## Abstract

Tuberculosis (TB) is one of the deadliest diseases, claiming ~2 million deaths annually worldwide. The majority of people in TB endemic regions are vaccinated with *Bacillus Calmette Guerin (BCG)*, which is the only usable vaccine available. BCG is efficacious against meningeal and disseminated TB in children, but protective responses are relatively short-lived and fail to protect against adult pulmonary TB. The longevity of vaccine efficacy critically depends on the magnitude of long-lasting central memory T (T_CM_) cells, a major source of which is stem cell-like memory T (T_SM_) cells. These T_SM_ cells exhibit enhanced self-renewal capacity as well as to rapidly respond to antigen and generate protective poly-functional T cells producing IFN-γ, TNF-α, IL-2 and IL-17. It is now evident that T helper Th 1 and Th17 cells are essential for host protection against TB. Recent reports have indicated that Th17 cells preserve the molecular signature for T_SM_ cells, which eventually differentiate into IFN-γ-producing effector cells. BCG is ineffective in inducing Th17 cell responses, which might explain its inadequate vaccine efficacy. Here, we show that revaccination with BCG along with clofazimine treatment promotes T_SM_ differentiation, which continuously restores T_CM_ and T effector memory (T_EM_) cells and drastically increases vaccine efficacy in BCG-primed animals. Analyses of these T_SM_ cells revealed that they are predominantly precursors to host protective Th1 and Th17 cells. Taken together, these findings revealed that clofazimine treatment at the time of BCG revaccination provides superior host protection against TB by increasing long-lasting T_SM_ cells.

## Author summary

Tuberculosis (TB) is one of the deadliest diseases, claiming ~2 million deaths annually worldwide. Bacillus Calmette Guerin (BCG) is the only usable vaccine available and exhibits efficacy against meningeal and disseminated TB in children. Consequently, the vast majority of people in TB endemic regions are vaccinated with BCG. However, host protective immune responses diminish over time due to gradual depletion of T central memory (T_CM_) cells, which are responsible for long-term host protection. Here, we provide evidence that revaccination with BCG along with the clofazimine, an approved drug for treatment of leprosy and drug-resistant TB, induces stem cell-like memory T (T_SM_) cells. T_SM_ cells are precursors to T_CM_ cells, and provide long-term host protection to TB by continuous supply of T_CM_ cells. Interestingly, these T_SM_ cells were generated from IL-17-producing T helper (Th)17 cells. These T_SM_ cells differentiated into T_CM_ and T effector memory (T_EM_) cells and maintained a stable pool of critically important Th1 and Th17 cells to provide optimal host protection against TB.

## Introduction

*Mycobacterium tuberculosis (M*.*tb)*, the etiological agent of tuberculosis (TB), causes nearly 2 million deaths annually worldwide. One third of the global population is infected with a latent form of TB, which represents an enormous reservoir for subsequent reactivation [1]. Conditions such as HIV infection that impair immunity may lead to TB reactivation [2]. In the absence of HIV co-infection, approximately 10% of latently infected individuals develop TB in their life time [2]. However, HIV co-infection increases this probability to more than 30% [3], either by directly eliminating CD4^+^ T helper (Th) cells or by modulating other host immune responses [4,5]. Although one third of the global population is latently infected with *M*.*tb*, the vast majority of individuals do not succumb to active TB, suggesting that immunity plays a role in host resistance. Th cells play a key role in host protective immunity against TB [6]. Previous reports, including studies from our group, have demonstrated that Th1 cells play an important role in host resistance against TB infection [7–10]. However, although the vast majority of TB patients mount Th1 responses, disease continues to progress in many patients [11]. These observations indicate that Th1 cell responses are essential but not sufficient for host protection. Recent reports have indicated that in addition to Th1 cells, Th17 cells play important roles in host protection, especially in secondary infection and vaccination [12,13]. However, Th2 and regulatory T (Treg) cells promote disease progression by counter-regulating these host-protective immune responses [8]. BCG induces sufficient Th1 responses but is ineffective in inducing optimal Th17 responses, which are essential for the generation of long-term memory responses [12,13]. Additionally, BCG induces antigen-specific Tregs and IL-4-producing Th2 cells, which may inhibit antigen-specific memory cells [14]. Previously, we and others reported that inhibition of Treg and Th2 responses along with BCG immunization enhances Th1 and Th17 cells, which promotes memory T cell responses that eventually translated into enhanced host protection [14].

Although BCG has limited efficacy against adult pulmonary TB, it is efficacious against disseminated and meningeal TB in young children [8]. Nevertheless, despite widespread vaccination with BCG, adult populations are not protected against pulmonary TB. It is well established that long-term memory responses are critically dependent on T central memory (T_CM_) cells rather than T effector memory (T_EM_) cells. Vaccine efficacy of *M*.*tb* depends upon the T_CM_:T_EM_ ratio [15,16]. T_EM_ cells are highly active and are responsible for antigen clearance, whereas T_CM_ cells are critical for long-term immunity and are capable of self-regeneration. An increase in the T_CM_ pool indicates reduced bacterial burden and enhanced long-term antigen-specific memory T cell responses [16]. It is highly likely that the limited protection of BCG against adult pulmonary TB is due to its inability to generate an adequate pool of *M*.*tb* antigen-specific T_CM_ cells. Although BCG induces some T_CM_ cells, these cells may decline over time, due to continuous exposure to environmental mycobacteria found in TB endemic regions [17–19]. Recently, a new memory T cell subset, T stem cell memory (T_SM_) cells, was characterized. These cells are endowed with stem cell-like properties, are capable of self-renewal, and exhibit the capacity to differentiate into a broad spectrum of memory subsets [20–23]. T_SM_ cells have been well documented among viral, parasitic and tumour-specific T cells [20–23]. Stemness of T_SM_ cells might be harnessed therapeutically to enhance the efficacy of vaccines for a variety of diseases. However, in TB, limited knowledge about T_SM_ cells is available.

Polyfunctional CD4^+^ T cells that produce IFN-γ, TNF-α , and IL-2 are critically important for protective immunity against TB [24,25]. One of the main challenges with the BCG vaccine is that chronic infection causes exhaustion of host-protective polyfunctional T cells, due to induction of various inhibitory receptors such as PD-1 and TIM-3 [26]. T cell exhaustion develops as a step wise loss of proliferation, cytokine production, and cytotoxic T lymphocyte (CTL) activity during chronic infection. Neutralizing these inhibitory molecules can restore activity and polyfunctionality of exhausted T cells to provide improved host protection [26].

The pathogenesis of TB contains two components: tissue destruction due to the infectious agent itself and tissue destruction due to the inflammatory process mediated by host immune responses. Thus, in some settings anti-inflammatory or immune modulatory agents can augment host protection when combined with conventional antibiotics [27]. Recently, we reported that the immune modulator clofazimine can enhance the vaccine efficacy of BCG by expanding antigen-specific T_CM_ cells in TB [28,29]. Clofazimine has been mainly employed for treatment of leprosy, and the WHO recently recommended its use as an antibiotic for drug-resistant TB [30]. Clofazimine is a lipophilic compound that binds with bacterial DNA and can enter host cells to interfere with the redox potential and to generate antimicrobial reactive oxygen species [31]. Clofazimine is also a pharmacological inhibitor of the potassium channel Kv1.3, which is predominantly expressed in T_EM_ cells, and Kv1.3 blockade by clofazimine increases T_CM_ populations [28]. Consistent with this observation, Kv1.3-deficient animals have an expanded T_CM_ population and these animals are resistant to T_EM_-dependent autoimmune encephalitis [32].

In keeping with these findings, and considering widespread BCG vaccination, we sought to examine if a booster dose of BCG supplemented with clofazimine can enhance BCG vaccine efficacy and provide long-term host protection against TB. We found that clofazimine increases the prevalence of T_CM_ and T_SM_ cells while having little effect on T_EM_ cells. Animals also exhibited markedly enhanced vaccine efficacy against a *M*.*tb* challenge. Therefore, inclusion of clofazimine during BCG revaccination enhances vaccine efficacy.

## Material and methods

### Ethics statement

All animal experiments were performed in accordance with the guidelines approved by the meeting of the Institutional Animals Ethics Committee, held at the Jawaharlal Nehru University (JNU), New Delhi, India (approval no IAEC Code no 19/2014), as well as at the International Centre for Genetic Engineering and Biotechnology (ICGEB), New Delhi, India (approval no ICGEB/IAEC/08/2016/TACF-JNU). These animal protocols were approved based on the recommendations from the national animal ethical committee of India and strictly follow the guidelines of the Department of Biotechnology of the Government of India. All mice used for experiments were ethically euthanized by asphyxiation in carbon dioxide, according to institutional and Department of Biotechnology (Government of India) regulations.

### Mice

Six- to eight-week-old C57BL/6 female mice and OT-II TCR transgenic mice were procured from the animal house facility of the JNU and ICGEB. All mice used for experiments were housed under barrier conditions in the Tuberculosis Aerosol Challenge Facility (TACF) of ICGEB and treated humanely according to specified animal care protocols of the TACF, ICGEB guidelines.

### BCG immunization experiments and drug administration

Mice were immunized subcutaneously with 1×10^6^ colony forming units (CFUs) of BCG (SBRI, Seattle, WA,USA) in 100 μl of sterile PBS. After 60 days of rest, these mice were again immunized with 0.5 × 10^6^ (CFUs) of BCG in 100 μL of sterile PBS. After 1 day of rest these mice were administered clofazimine intra-peritoneally at 5 mg/kg of body weight and Isoniazid (INH) intra-peritoneally at 25mg/kg in 100 μL of phosphate-buffered saline (PBS) once a week for a total of 4 weeks. Mice were subsequently rested for 30 days. These mice were then challenged via the aerosol route with *M*.*tb* strain H37Rv, as described below, and organs were harvested after euthanasia to determine bacterial burden and immune phenotypes at different time points. For *in vitro* studies the concentration of clofazimine and INH were 20 μg/ml ***respectively*** and ovalbumin were 50 μg/ml,.

### *M*. *tuberculosis* low-dose aerosol infection of mice

*M*.*tb* strain H37Rv (ATCC 27294; American Type Culture Collection) was a kind gift from the Colorado State University. Mouse infections were performed in accordance with the low-dose aerosol infection model, using a Madison aerosol chamber (University of Wisconsin, Madison) with the nebulizer pre-calibrated at approximately 90–120 CFUs per mouse. *M*.*tb* strain H37Rv was grown to mid–log phase (optical density at 600 nm, approximately 0.6) in Middlebrook 7H9 media with 0.1% Tween 80 (Sigma), 0.2% glycerol and 10% Middlebrook Oleic Acid, albumin, dextrose, and catalase enrichment medium (OADC, Difco^™^, USA). Bacteria were stored at -80°C in 20% glycerol stocks for further experiments. For aerosol infection, cultures were washed twice with PBS and made into a single-cell suspension by passing through a 26-gauge needle for 10 times. Next, 15 ml of the *M*.*tb* H37Rv single-cell suspension (100×10^6^ cells) was placed in the nebulizer reservoir of the Madison Aerosol Chamber, calibrated to deliver the desired CFUs of bacteria into the lungs of mice kept in the chamber in 15-minute cycles. At 24 hours after aerosol challenge 3 mice were euthanized for quantitation of pathogen delivery to lungs by measurement of CFUs in lung homogenates. Mice were found to be infected with approximately 110–120 CFUs of *M*.*tb* in their lungs. The mice were maintained in a biosafety containment facility.

### Quantitation of pathogen burden by CFU counts

Randomly selected mice were euthanized at various time points, after which organs were harvested, homogenized in 0.2-μm–filtered PBS containing 0.05% Tween 80, and plated onto 7H11 Middlebrook plates containing 10% OADC enrichment medium (Difco^™^, USA). Lung and spleen cell homogenates were plated on 7H11 plates and incubated at 37°C for approximately 21 days. CFUs were counted and the pathogen burdens in lungs and spleens were estimated.

### Flow cytometry

Spleens and lungs were isolated from mice and macerated by frosted slides in ice-cold RPMI-1640 medium (Gibco; Invitrogen) containing 10% foetal bovine serum to prepare a single-cell suspension. Red blood cells were lysed with lysis buffer by incubating at room temperature for 1–2 minutes, and then washed with RPMI-1640 medium. These fresh lymphocytes were counted and cultured overnight in the presence of H37Rv complete soluble antigen (CSA). For surface staining 1×10^6^ cells per well were cultured in 12-well-plates. Before staining cells were washed twice with FACS buffer and then stained with antibodies directed against surface markers. For surface staining lymphocytes were stained with monoclonal antibodies. After adding antibodies, cells were kept for 40 minutes at room temperature. Then cells were washed again with buffer and re-suspended in FACS buffer. For intracellular staining 2×10^6^ cells were cultured per well and stimulated with H37Rv complete soluble antigen overnight, with 1 μg/ml of brefeldin A (Bio-legend) were added during the last 6 hours of culture. For intracellular staining, surface-stained cells were fixed with 100μl of fixation buffer (Bio-legend) for 30 minutes, and then washed twice with permeabilization buffer (Bio-legend), re-suspended in 200 ***μl*** of permeabilization buffer, and stained with fluorescently labelled antibodies. After staining cells were washed again with FACS buffer and re-suspended in FACS buffer. The fluorescence intensity of fluorochrome-labelled cells was measured by flow cytometry (FACS-LSR Fortessa; BD Biosciences). BD FACS-DIVA Software was used to acquire the cells, and data analysis was performed using FlowJo software (Tree Star). To determine double positive (IFN-γ and TNF-α, IL-2 and IFN-γ, or TNF-α and IL-2 producing cells from the CD4^+^ T_CM_ or T_EM_ compartment) and triple positive cells (IFN-γ, TNF-α and IL-2 producing cells from the CD4^+^ T_CM_ and CD4^***+***^ T_EM_ compartment) standard Boolean algorithm was used [33].

### Antibodies and reagents

We used antibodies against the following markers: CD3 (BD; clone145-2C11), CD4 (Bio legend, clone GK1.5; or FITC, clone RM4-5), CD8 (Bio legend; clone 536.7), CD44 (Bio legend clone, IM7), CD62L (Bio legend clone MEL-14), CD25 (Bio legend clone 3C7), IFN-γ (Bio legend cloneXMG1.2), IL-2 (Bio legend clone JES6-5H4), IL-6 (Bio legend clone MPS-20F3), IL-17 (Bio legend clone tc11-18h10.1), IL-4 (Bio legend clone11b11), TNF-α (Bio legend clone MP6XT22), TGF-β (Bio legend clone TW7-16B4), CD45RA (BD, clone 14.8), CD95 (BD, Clone Jo2), CD122 (BD, clone TM-β), CD127 (BD, Clone SB/199), PD-1 (Bio legend clone, 29F.1A12), Tim-3 (Bio legend, clone B8.2C12), Foxp3 (Bio legend, Clone MF-14). OADC was from DIFCO, and RPMI-1640 and FBS were obtained from Gibco-BRL, ovalbumin from Sigma, and clofazimine from Sigma.

### Cytokine assay

Cytokines in the culture supernatant were assayed by a Luminex microbead-based multiplexed assay using commercially available kits according to the manufacturer's instructions (BioPlex, Bio-Rad, Mouse Cytokine Group-1). Samples were run on Bio-Rad Bio-plex 200 system.

### q-PCR

Real-time quantitative RT-PCR analysis was performed using Bio-Rad Real-Time thermal cycler (Bio-Rad, USA). The reaction was set up according to the manufacturer’s protocol. Enzyme reverse transcriptase and SYBR green Master mix were procured from Bio-Rad (USA). The relative expression level of mRNAs was normalized to that of internal control GAPDH by using 2-ΔΔCt cycle threshold method. The primer sequences were as follows: *tcf7*: Forward: TACTATGAACTGGCCCGCAAG, Reverse: CCTGTGGTGGATTCTTGATG; *IL-17*: Forward: TACCTCAACCGTTCCACG Reverse: TTTCCCTCCGCATTGACACA; *t-bet* Forward, GCCAGGGAACCGCTTATATG; Reverse GACGATCATCTGGGTCACATTGT,

*ROR*γ*t*: Forward: CTGCTGAGAAGGACAGGGAG, Reverse: AGTTCTGCTGACGGGTGC, and *GAPDH*: Forward: AAGGGCTCATGACCACAGTC, and Reverse: CAGGGATGATGTTCTGGGC.

### Histological studies

Pieces of lungs from mice at 60 days post infection were perfused, removed, and fixed in 4% paraformaldehyde solution for 12 hrs, then transferred to 70% ethanol, paraffin blocks were made, 6 μm sections were cut, and stained with haematoxylin and eosin according to our previous work. Similarly, AFB staining was performed [34].

### *In vitro* experiments with peritoneal macrophages

Six- to eight-week-old C57BL/6 mice were given an i.p. injection of 1 ml thioglycollate medium (4%). After 5 days macrophages were harvested from peritoneal lavage. Macrophages were washed once with cold PBS and suspended in cold RPMI-1640. Cells were counted, seeded on 12-well plates and maintained at 37ºC in RPMI-1640 medium supplemented with penicillin–streptomycin (1,000 units/ml) and 10% heat-inactivated fetal calf serum. After overnight incubation, non-adherent cells were washed. Adherent cells were treated with CSA (20 μg/ml) followed by clofazimine treatment. Cells were then kept at 37°C in a CO_2_ incubator. After 48 hrs of treatment macrophages were harvested for flow cytometry.

### Co-culture experiments with T cells from OT-II mice

Treated and untreated macrophage (described in the previous section) were co-cultured with CD4^+^ T cells at 1:3 ratios. CD4^+^ T cells were purified from splenocytes of OT-II transgenic mice by using nylon wool fiber (Pro assay). For determining ovalbumin-specific immune responses cells were stimulated with ovalbumin at a concentration of 50 μg/ml. After 48 hrs of co-culture these cells were harvested for flow cytometry.

### Spectrophotometry analysis

Clofazimine has peak absorption spectra at 450 nm. At different time points (5 days and 31 days) after clofazimine treatment, the residual amounts of clofazimine in the serum of mice were determined using a UV spectrophotometer (VARIAN) [35].

### Determination of residual clofazimine in fat bodies

Clofazimine shows maximum bioavailability in the fatty tissue. At different time points (5 days and 25 days) after clofazimine treatment the residual amounts of clofazimine in the fatty tissue of mice were determined by direct visualization and spectroscopy. Control and clofazimine-treated mice were sacrificed and their subcutaneous and mesentric fat bodies were visualized. After taking equal weights of fat tissue (10 mg) from different groups of mice, clofazimine was extracted as described by Baik et al. [36]. These extracted products were suspended into methanol and absorption spectra were determined by UV spectrophotometry (VARIAN).

### Statistical analysis

All data were derived from ≥3 independent experiments, and each group included at least 5 mice in each experiment. Statistical analyses were conducted using Graph pad software and values are presented as means with standard deviations. Significant differences between the group means were determined by analysis of variance followed Tukey’s Post hoc test. Differences were considered statistically significant at P≤0.05.

## Results

### Clofazimine enhances the efficacy of BCG revaccination

**Fig 1:**
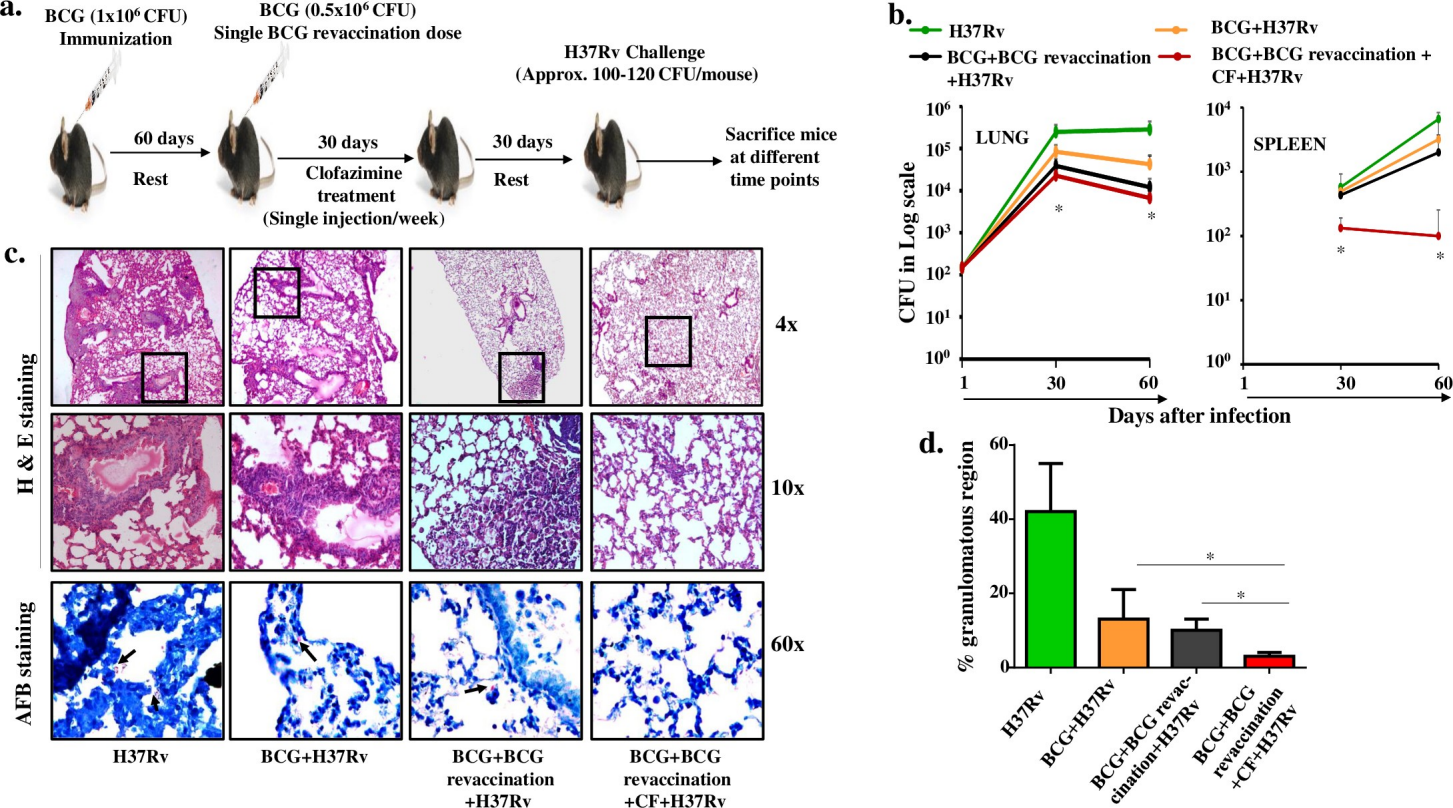
Clofazimine enhances the capacity of BCG revaccination to protect against TB. (a) Schematic representation of the experiment. Mice were immunized with BCG and rested for 60 days. Then, a single BCG booster dose was given followed by clofazimine treatment (1 injection/week) for one month, and finally animals were rested for another month. Mice were then challenged with H37Rv via the aerosol route, with a low-dose of inoculum of approximately 100–120 colony-forming units (CFUs) per mouse. After that mice were euthanized at various time points (30 or 60 days), lungs and spleen were harvested and assessed for bacterial burden and immunological parameters. (b) CFU counts of lungs and spleen at different time intervals. (c) Haematoxylin & Eosin staining of lung sections showing granulomatous regions and AFB staining of lung sections for mycobacteria. Arrows depict the presence of *M*.*tb*. (d) Relative granulomatous regions in different experimental groups. All data are representative of 3 independent experiments and each group included at least 5 mice in each experiment. All values are represented as Mean±SD. Statistical analyses were performed by ANOVA with Tukey’s post hoc analysis. * denotes P ≤ .05. CF, clofazimine.

BCG immunization is mandatory for newborn children in India and other South Asian countries, which are endemic for *M*.*tb* infection. BCG-induced host protective responses diminish over time, which might be due to human and mycobacterial genetics, different coinfections, or continuous exposure to environmental mycobacteria that might cause exhaustion of the long-term memory T cells [37]. Restoring these memory T cell pools may therefore enhance protection against TB. Since people in these endemic countries are already vaccinated with BCG, we considered the possibility of administering a booster dose of BCG (BCG revaccination) in a context that improves host immune responses. Clofazimine is a well-known antibiotic and is currently used for treatment of leprosy [38,39]. Recently, clofazimine was approved for treatment of drug-resistant TB. Interestingly, clofazimine is also an excellent immune modulator that enhances T_CM_ responses while reducing T_EM_ responses [28], and exhibits efficacy against autoimmune diseases such as experimental autoimmune encephalomyelitis [32]. In some settings of long term dosing clofazimine exhibited bioavailability up to 70 days [40], but a single dose or short 8-day treatment regimen displayed bioavailability of 8 to 10 days [41,42]. Despite its long bioavailability and tissue deposition, Swanson et al. established that only free clofazimine in serum has active antimicrobial activity and that clofazimine precipitated in tissues as crystals or bound with proteins in tissues or even serum is devoid of antimicrobial properties [40]. Based on these findings we considered whether clofazimine can enhance the efficacy of a booster dose of BCG in previously immunized mice. Animals were subcutaneously immunized with BCG and rested for 60 days, and these animals were subsequently provided with a booster dose of BCG (0.5x10^6^ CFU) and treated with four doses of clofazimine in weekly intervals. We selected a one week gap between each dose to ensure sufficient bioavailability. These animals were then rested for an additional 30 days followed by aerosol challenge with a low dose of the virulent *M*.*tb* strain H37Rv (**Fig 1A**). Bacterial burdens in lungs and spleens were determined at various time points after challenge. As expected, we observed that clofazimine-treated animals had dramatically reduced CFUs in both lungs and spleens at all time points (**Fig 1B**). However, dissemination of TB bacteria from lungs to other organs was more restricted in clofazimine-treated and BCG-revaccinated animals, which was expected, as bacterial dissemination is slow, at which time adequate antigen-specific adaptive immune responses that effectively clear the bacteria are already present. Histological analyses of lungs revealed that clofazimine treatment significantly reduced granulomatous regions (**Fig 1C and 1D)**. We found that *M*.*tb* infection induced extensive granulomatous regions throughout the lung sections, whereas infection in BCG-vaccinated animals and mice that received a primary and booster BCG vaccination had reduced histopathology. Importantly, animals that received clofazimine at the time of BCG revaccination exhibited more profoundly reduced pathology with few granulomatous regions (**Fig 1C**). Acid Fast Bacillus (AFB) staining of histological sections confirmed these findings, as mice treated with clofazimine had reduced numbers of bacteria as compared with untreated animals (**Fig 1C, lower panels). *To*** ascertain that the observed improvement in vaccine efficacy in clofazimine-treated animals was not due to the residual clofazimine that exhibits direct anti-microbial activity, we analysed clofazimine content in serum and fatty tissues of animals. We could not detect clofazimine in the serum at 31 days after clofazimine treatment **(S1 Fig),** and only residual amounts were present in fat tissues at 5 days after clofazimine treatment ***(*S2 Fig).** Therefore, the observed vaccine efficacy is most likely due to the improved immune responses induced by clofazimine.

### Clofazimine enhances long-term memory responses of BCG revaccination

**Fig 2:**
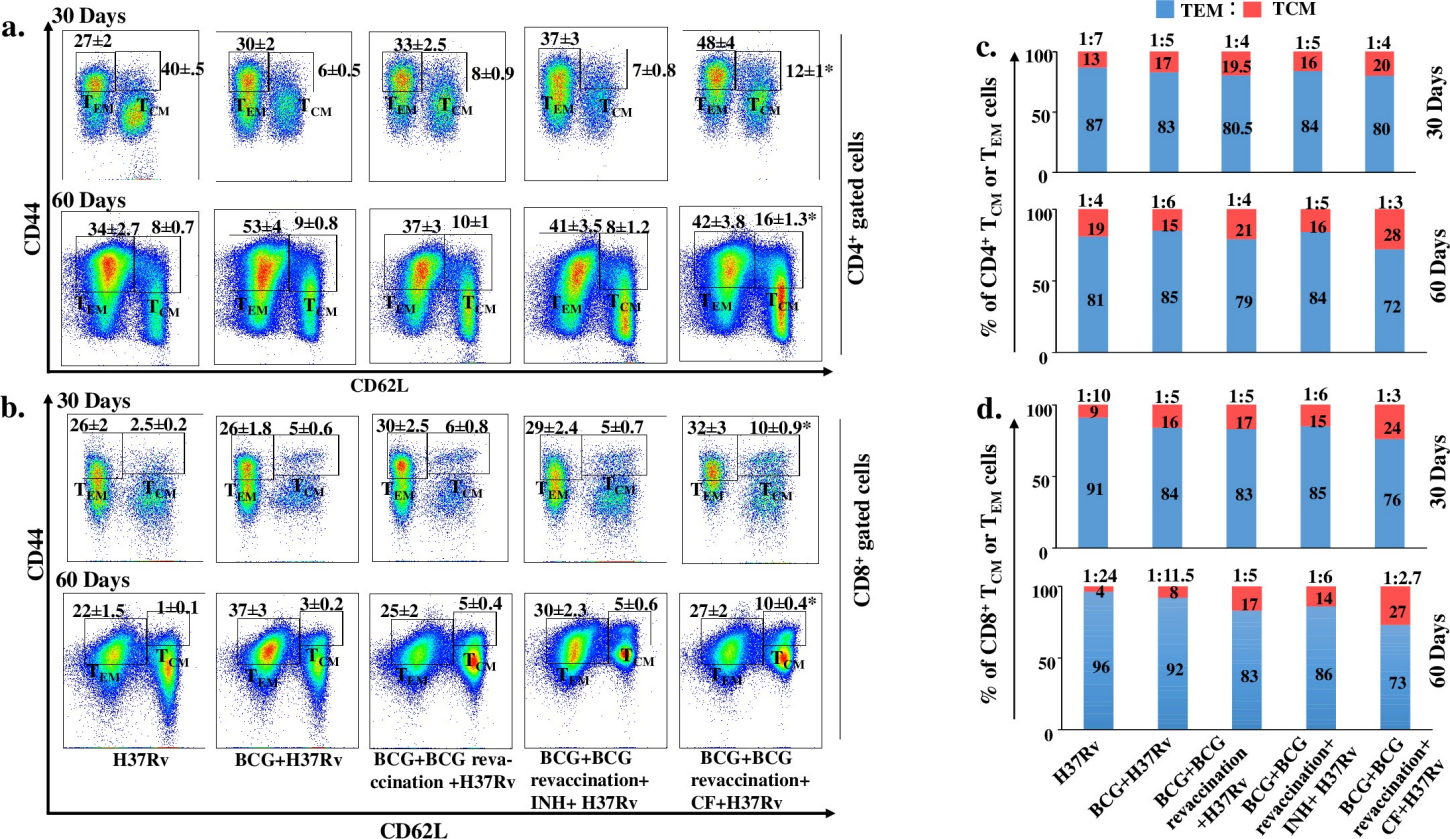
Clofazimine enhances the capacity of BCG revaccination to optimize the T_CM_: T_EM_ cell ratio. Spleens were harvested at different time points after aerosol challenge. Single cell suspensions were made. Cells were cultured overnight with *M*.*tb* lysate (CSA), stained with, anti-CD4, -CD8, -CD44 and -CD62L antibodies and analysed by flow cytometry. (a) T_EM_ and T_CM_ populations among CD4^+^ cells at different time points. (b) T_EM_ and T_CM_ populations among CD8^+^ cells at different time points. (c) Pattern of changes in the percentage of CD4^+^ T_EM_ and T_CM_ cell populations at different time points. (d) Pattern of changes in the percentage of CD8^+^ T_EM_ and T_CM_ cell populations at different time points. All data are representative of 3 independent experiments and each group included at least 5 mice in each experiment. All values are represented as Mean±SD. Statistical analyses were performed by ANOVA with Tukey’s post hoc analysis. In figure a and b comparisons of T_CM_ cells were done between the BCG+BCG revaccination+CF+H37Rv group and all other experimental groups. * denotes P ≤0.05. INH, Isoniazid; CF, clofazimine.

In TB endemic regions T_CM_ cells are drastically exhausted in BCG-vaccinated individuals, for reasons that remain unknown. Nevertheless, it is likely that continuous exposure of environmental mycobacteria might impair the host-protective T_CM_ cell population [17–19]. Moreover, a major failure of the BCG vaccine is its inability to induce high-quality T_CM_ cells [16]. Long-term vaccine efficacy primarily depends upon the balance between T_CM_ and T_EM_ cells generated during immunization [43]. To obtain insight into the effects of clofazimine on memory T cells, we examined the kinetics of T_CM_ and T_EM_ cells in lungs and spleen. In this experiment we also included BCG revaccination with INH treatment as a control group, as INH is one of the important anti-TB drugs. We harvested the spleen at various time points and analysed T_CM_ (CD44^hi^CD62L^hi^) and T_EM_ (CD44^hi^CD62L^low^) cells in both the CD4^+^ (**Fig 2A**) or CD8^+^ T cell subsets (**Fig 2B**). At 30 and 60 days post infection the number of splenic T_CM_ cells increased significantly in animals that received clofazimine during BCG revaccination in both the CD4^+^ and CD8^+^ T cell lineages, as compared with animals that received BCG vaccination, BCG-revaccination, or BCG-revaccination with INH treatment (**Fig 2A and 2B**). As time progressed from 30 to 60 days the T_CM_:T_EM_ ratio of CD4^+^ T cells increased significantly from 1:4 to 1:3 in animals that received clofazimine during BCG revaccination. In contrast, for animals that received only BCG-revaccination or BCG-revaccination with INH treatment the T_CM_:T_EM_ ratio remained stable at 1:4 or 1:5, respectively, at both 30 and 60 days post infection. Moreover, in BCG-immunized animals the T_CM_:T_EM_ ratio decreased over time, from 1:5 (day 30) to 1:6 (day 60) **(Fig 2C**). In CD8^+^ T cells, the T_CM_:T_EM_ ratio showed a similar trend as time progressed **(Fig 2D)**. However, in BCG-immunized animals the reduction in the T_CM_:T_EM_ ratio was more prominent in CD8^+^ T cells compared with CD4^+^ T cells (**Fig 2C and 2D**). In this experiment we employed INH to test whether the antimicrobial properties of drugs have any role in immunomodulation. We found that INH did not exhibit any major immunomodulatory properties, whereas clofazimine increased the efficacy of BCG revaccination by modulating the host immune system. Moreover, to confirm our findings we also determined the in vitro immune modulatory properties of clofazimine and INH in splenic cultures of infected animals. Surprisingly, we found that clofazimine dramatically increased the T_CM_ vs T_EM_ ratio as well as IFN-γ- and IL-17-producing CD4^+^ cells (**S3 Fig**). In contrast, INH did not show any immunomodulatory effects. We therefore concluded that clofazimine has substantial immune modulatory effects and that under our experimental conditions it did not have any direct anti-bacterial effects. We also measured IL-7 cytokine receptor α (CD127) expression, as CD127 is responsible for stable maintenance or survival of the T_CM_ cell lineage [44]. We found that clofazimine-treated mice expressed higher levels of CD127 on their T_CM_ cells than the other experimental groups (**S4 Fig**). We also examined the status of memory T cells in lungs at 60 days post infection and found a similar trend as that of spleen (**S5 Fig**). Thus, clofazimine treatment during a booster BCG immunization modulates T lymphocyte memory responses to increase the T_CM_:T_EM_ ratio, which might provide long-term protection against TB.

### Clofazimine enhances polyfunctional T_CM_ and T_EM_ cells during BCG revaccination

Memory T cells exhibit profound functional diversity, which depends on a variety of factors, including their differentiation state, tissue homing capacity, long-time survival and expression of cytokines (IFN-γ, TNF-α and IL-2). Production of these three major cytokines (IFN-γ, TNF-α and IL-2) by polyfunctional T cells is critical for protection against TB [45].

**Fig 3:**
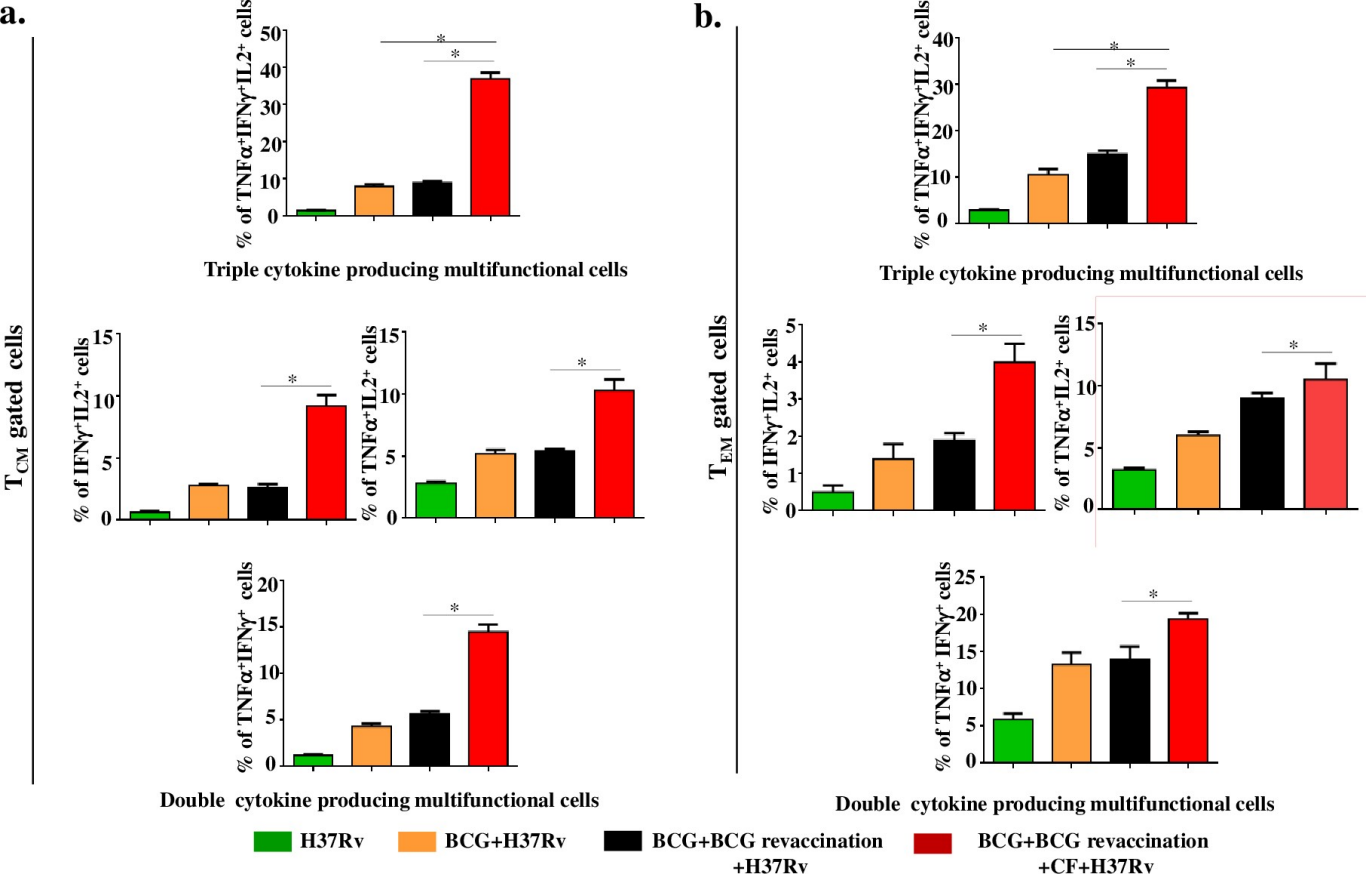
Clofazimine enhances the capacity of BCG revaccination to increase the percentage of polyfunctional CD4^+^ T cells. Spleens were harvested and single cell suspensions were made. Cells were cultured overnight with *M*.*tb* lysate (CSA) and stained with, anti-CD4, -CD44, -CD62L, -TNF-α, -IFN-γ, and -IL-2 antibodies. (a) Percentage of cytokine-producing CD4^+^ T_CM_ cells. (b) Percentage of cytokine-producing CD4^+^ T_EM_ cells. All data are representative of 3 independent experiments and each group included at least 5 mice in each experiment. All values are represented as Mean±SD. Statistical analyses were performed by ANOVA with Tukey’s post hoc analysis. * denotes P ≤0.05. CF, clofazimine.

We correlated the functional status of memory T cells by determining IFN-γ, TNF-α and IL-2-producing cells among the T_CM_ and T_EM_ cell compartments of the different experimental groups of animals. T_CM_ cells isolated from animals that received clofazimine during BCG revaccination exhibited significantly (P ≤0.05) increased levels of TNF-α^+^IFN-γ^+^IL-2^+^ triple-positive (37%), TNF-α^+^IFN-γ^+^ (14%), IFN-γ^+^IL-2^+^ (9%) and TNF-α^+^IL-2^+^ (10%) double-positive T cells as compared with all other groups (**Fig 3A**). In contrast, animals receiving only BCG revaccination showed significantly increased (P ≤0.05) levels of IFN-γ^+^TNF-α^+^ double-positive (6%) T_CM_ cells as compared with H37Rv infected and single BCG vaccinated animals (1,4%), respectively (**Fig 3A**). However, for T_EM_ cells, mice receiving BCG revaccination with clofazimine showed significantly (P ≤0.05) increased levels of TNF-α^+^IFN-γ^+^IL-2^+^ triple-positive (29%) and IFN-γ^+^IL-2^+^ (4%), TNF-α^+^IFN-γ^+^ (19%), and TNF-α^+^IL-2^+^ 10%) double-positive T cells as compared with BCG-revaccinated animals (15%, 2%, 14% and 9%, respectively) (**Fig 3B**). Nonetheless, the magnitude of differences for triple-positive cells and IFN-γ^+^IL-2^+^ double-positive cells among T_CM_ cells (9%) was higher than among T_EM_ cells (4%). However, the magnitude of TNF-α^+^IFN-γ^+^ double-positive cells (19%) was higher among T_EM_ cells. These results indicated that mice receiving BCG revaccination with clofazimine showed enhanced IFN-γ^+^TNF-α^+^IL-2^+^ triple-positive cells in both the T_CM_ and T_EM_ populations (**Fig 3A and 3B**).

It is well known that M.tb antigen-specific polyfunctional effector T cells are exhausted due to induction of inhibitory receptors such as PD-1 and Tim-3 [26]. As supplementation of clofazimine during BCG revaccination induces higher levels of antigen-specific effector T cells, we determined whether these cells have an exhausted phenotype. Remarkably, expression of these molecules by both CD4 and CD8 cells in animals that received a combination of BCG revaccination plus clofazimine was lower as compared with any of the other experimental groups **(S6 Fig).** This observation suggested that the antigen-specific T cells induced following BCG revaccination and clofazimine treatment are not exhausted and are functionally active.

### Clofazimine induces stem cell-like memory T cells during BCG revaccination

**Fig 4:**
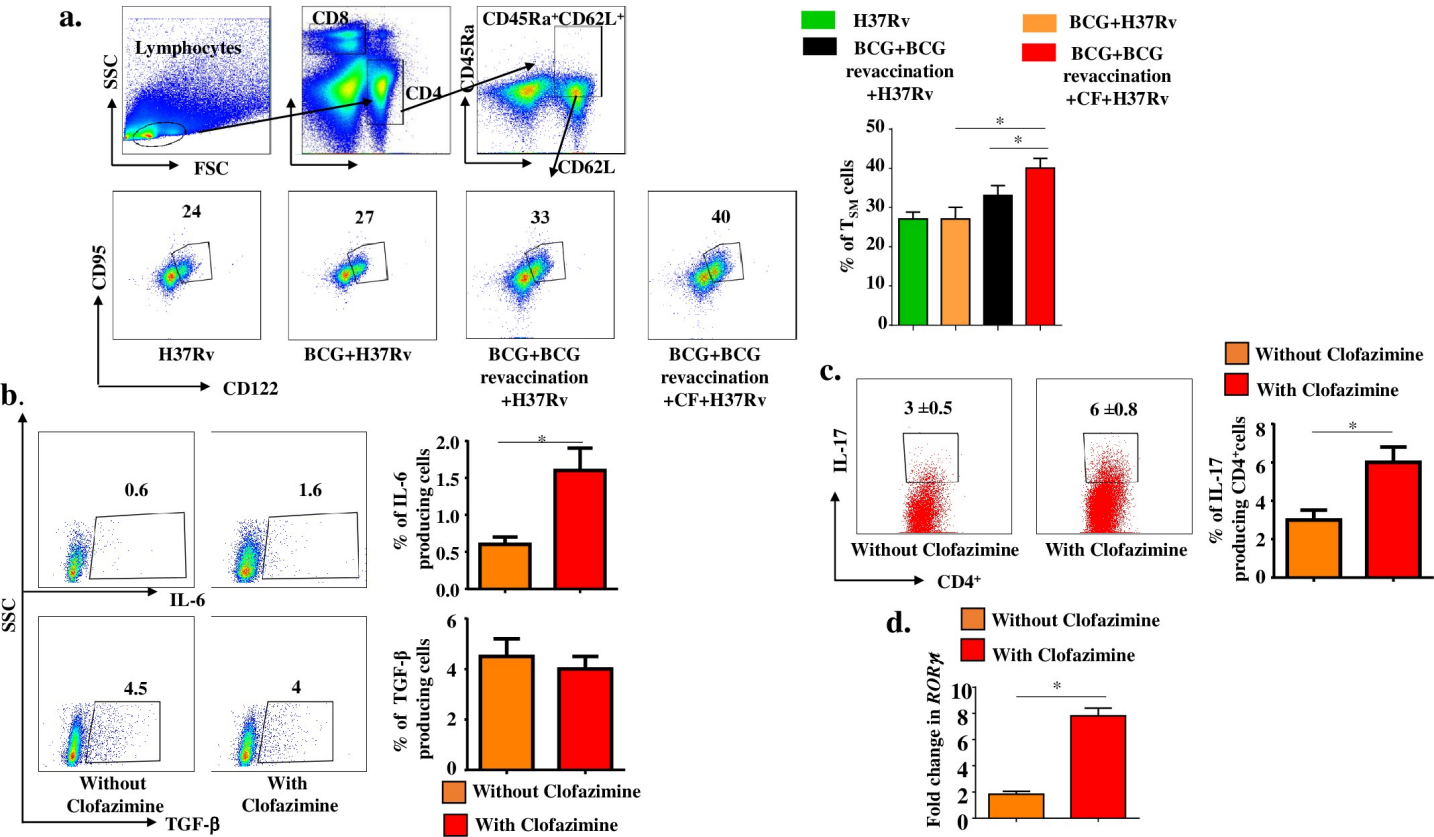
Clofazimine enhances the capacity of BCG revaccination to increase the frequency of T_SM_ cells. Spleens were harvested and single cell suspensions were made. Cells were cultured overnight with *M*.*tb* lysate (CSA) and stained with, anti-CD4, -CD62L, -CD45RA, -CD95 and -CD122 antibodies. (a) Representative gating strategy for isolation of CD4^+^CD45RA^+^CD62L^+^CD95^+^CD122^+^ T_SM_ cells from the lymphocyte population and percentage of T_SM_ cells in different experimental groups. (b) Percentage of IL-6- and TGFβ-producing peritoneal macrophages cultured in the presence or absence of clofazimine. (c) Peritoneal macrophages were primed with *M*.*tb* antigen in the presence or absence of clofazimine for 4 hrs. Then, the primed macrophages were cocultured with T cells from OT-II transgenic mice in the presence of ovalbumin overnight. Finally, the percentage of IL17-producing CD4^+^ cells was analysed and (d) expression of *ROR*γ*t* was determined by qPCR analysis. All data are representative of 3 independent experiments and each group included at least 5 mice in each experiment. For in vitro experiments at least 3 biological replica and 3 technical replica were performed. All values are represented as Mean±SD. Statistical analyses were performed by ANOVA with Tukey’s post hoc test. * denotes P ≤0.05. CF, clofazimine.

Recently, a new memory T cell subset, T stem cell memory (T_SM_) cells, was discovered with improved capability of regeneration, differentiation, as well as long-term stability [20–23]. This subset of T cells is thought to be the progenitor of T_CM_ and T_EM_ cells [20]. T_SM_ cells typically express CD45RA and CCR7 and thus phenotypically resemble naïve T cells, but their co-expression of stem cell markers, such as CD95 and CXCR3, distinguishes them from naïve T cells [20], and these cells also express CD122 (IL-2 receptor β), which promotes their self-renewal [22,46]. Antigen-specific T_SM_ cells have been well characterized in viral, parasitic and tumour-specific CD8^+^ T cells [20–23], and a recent report described *M*.*tb*-specific CD4^+^ T_SM_ cells as the source of T_CM_ and T_EM_ cells [29]. To examine these cells, we first identified CD4^+^CD62L^+^CD45RA^+^ naïve T cells and subsequently analysed expression of CD95 and CD122 (**Fig 4A**). We found a significant increase (P ≤0.05) in CD4^+^ T_SM_ (CD62L^+^CD45RA^+^CD95^+^CD122^+^) cells in animals receiving BCG revaccination and clofazimine (40%) compared with mice receiving only BCG vaccination (27%) or revaccination (33%) (**Fig 4A**). Unstained control of CD122 and CD95 are shown separately with all other unstained control of different experiments **(S7 Fig).**

It has been recently reported that IL-17 is one of the key players in memory T cell generation. IL-17 promotes T_SM_ cell generation by activating the Wnt/β-catenin signalling pathway, which restricts antigen-specific T cells to the naïve compartment and ultimately shifts them towards T_SM_ cells [47]. We examined if clofazimine has a role in the generation of IL-17-producing T cells. A combination of IL-6 and TGF-β is required for the differentiation of IL-17-producing T cells [48]. We tested if clofazimine has any influence on the production of these cytokines in macrophages. We challenged macrophages with complete soluble antigen (CSA), treated them with clofazimine, and via intracellular staining assessed the cytokines IL-6 and TGF-β. We found that clofazimine treatment increased the percentage of IL-6-producing cells, whereas levels of TGF-β were almost unchanged by clofazimine treatment **(Fig 4B)**. Furthermore, when these clofazimine-treated and untreated macrophages were co-cultured with T cells isolated from OT-II TCR transgenic mice in the presence of ovalbumin, we found a subtle increase in IL-17-producing transgenic T cells (**Fig 4C**) expressing high levels of the transcription factor RORγt (**Fig 4D**) in the clofazimine-treated group. Therefore, our results suggest that clofazimine treatment creates a niche for the generation of IL-17-producing T helper 17 cells that promote the differentiation of T_SM_ cells.

### Clofazimine enhances IFN-γ and IL-17 responses during BCG revaccination

**Fig 5:**
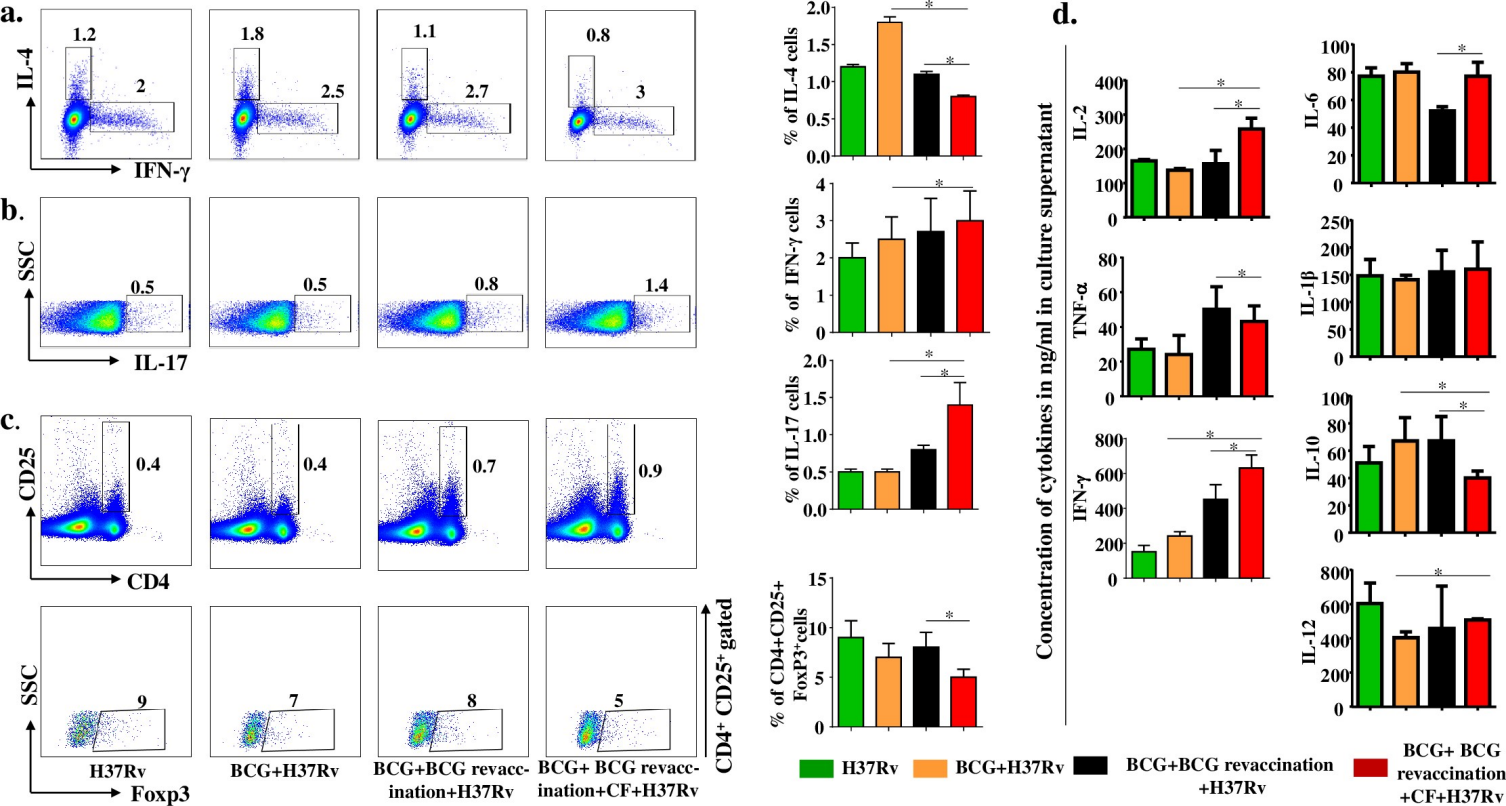
Clofazimine enhances the capacity of BCG revaccination to increase the prevalence of antigen-specific Th1 and Th17 cells. Spleens were harvested and single cell suspensions were made. Cells were cultured overnight with *M*.*tb* lysate (CSA), supernatants were collected for cytokine multiplex assay and cells were stained with,anti-CD4,CD25 -IL-4, -IFN-γ, -IL-17 and Foxp3 antibodies and analysed by flow cytometry. (a) Schematic representation of FACS data and bar graphs for percentage of IL-4- and IFN-γ-producing CD4^+^ cells in different experimental groups. (b) Schematic representation of FACS data and bar graphs for percentage of IL-17-producing CD4^+^ cells in different experimental groups. (***c***) Schematic representation of FACS data and bar diagrams for the percentage of CD4^+^CD25^+^Foxp3^+^ cells in different experimental groups. (***d***) Concentration of different polarizing cytokines measured by multiplex assay. All data are representative of 3 independent experiments and each group included at least 5 mice in each experiment. All values are represented as Mean±SD. Statistical analyses were performed by ANOVA with Tukey’s post hoc test. * denotes P ≤0.05. CF, clofazimine.

It is well known that Th1 responses provide host protection against *M*.*tb* infection. However, Th17 responses are also required for protection against secondary infection and generation of long-term memory responses [49,50]. Therefore, we quantified different CD4^+^ T cell subsets based on their signature cytokine production in our experimental groups at 60 days post infection. We found that IFN-γ and IL-17 producing cells were significantly increased (**Fig 5A and 5B**), whereas IL-4 producing Th2 cells were reduced in animals that received BCG revaccination and clofazimine treatment (**Fig 5A**). This observation prompted us to measure the Treg population. We found that the Treg population (CD4^+^CD25^+^Foxp3^+^ cells) was also significantly decreased (**Fig 5C**). Together, these data indicated that clofazimine treatment increases the protective IFN-γ and IL-17 immune response, which promotes memory T cell generation. We also measured cytokines from splenocyte culture supernatants and found that clofazimine treatment significantly enhanced the levels of IL-2, TNF-α, IFN-γ, IL-12, and IL-6 and decreased the levels of IL-10. Moreover, there was a stable maintenance of IL-1β among the animals receiving different treatments (**Fig 5D**). Enhancement in the levels of IL-2, TNF-α, and IFN-γ **(Fig 5D)** corroborated the finding that clofazimine treatment enhances the levels of polyfunctional memory T cells. Moreover, the increased level of IL-6 **(Fig 5D)** suggested that clofazimine treatment establishes a cytokine milieu that promotes Th17 cell generation.

### Clofazimine promotes conversion of IL-17-producing Th17 cells into T_SM_-like cells

**Fig 6:**
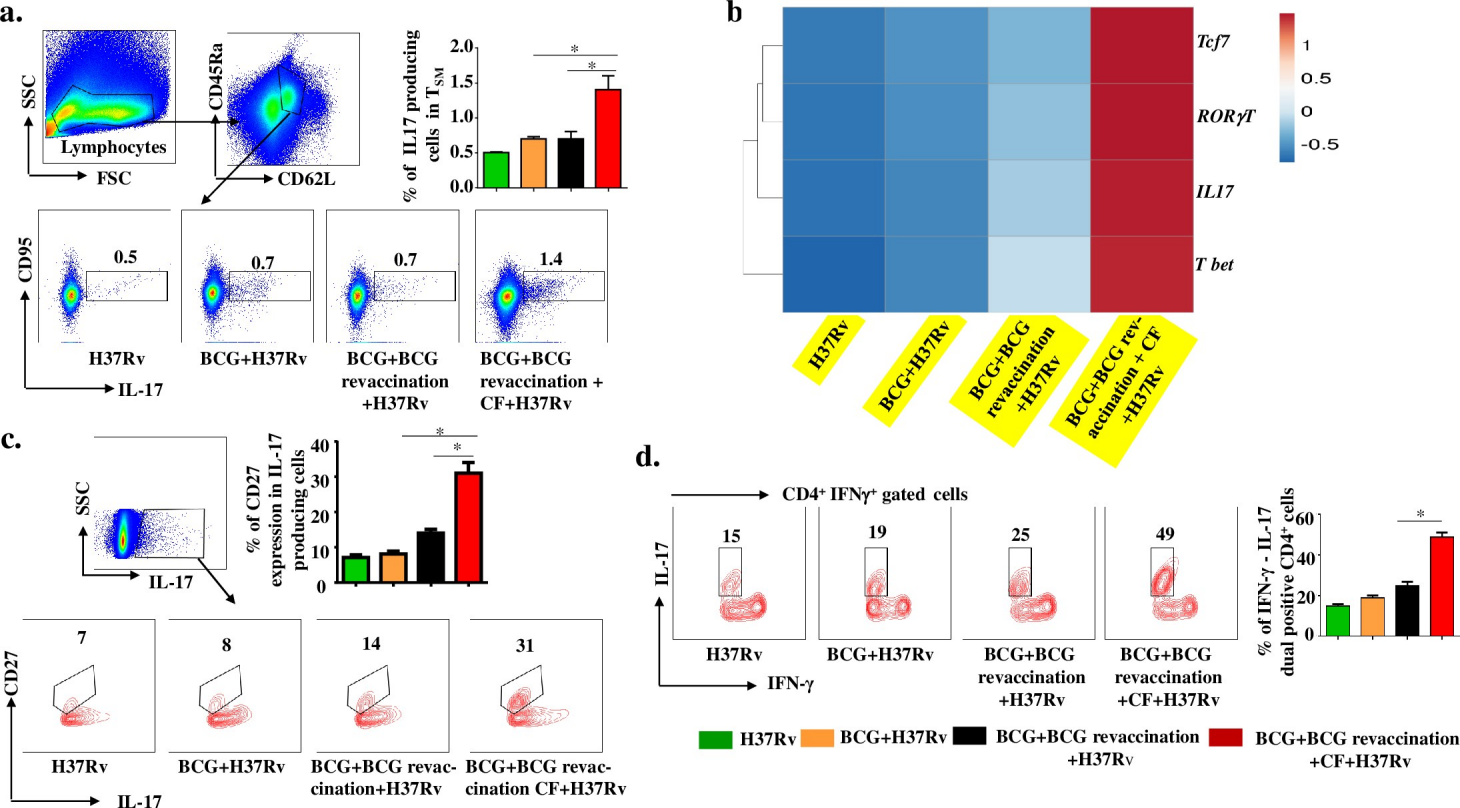
Clofazimine enhances the capacity of BCG revaccination to increase the conversion of IL-17-producing T cells into T_SM_ and IFN-γ-producing T cells. Spleens were harvested and single cell suspensions were made. Cells were cultured overnight with *M*.*tb* lysate (CSA), then stained with, anti-CD4, -CD62L, -CD45RA, -CD95 and -IL-17 antibodies and analysed by flow cytometry. (a) Schematic representation of FACS data and bar diagrams for IL-17^+^ cells with a T_SM_ signature in different experimental groups. (b) Expression of *tcf-7*, *ROR*γ*t*, *t-bet* and *IL-17* genes in different experimental groups. (c) Percentage of IL-17^+^CD27^+^CD4^+^ cells in different groups of animals. (d) Percentage of IL-17^+^IFN-γ^+^CD4^+^ cells in different groups of animals. All data are representative of 3 independent experiments and each group included at least 5 mice in each experiment. All values are represented as Mean±SD. Statistical analyses were performed by ANOVA with Tukey’s post hoc test. * denotes P ≤0.05. CF, clofazimine.

Recently, it has been shown that Th17 cells transform into less-differentiated, naïve-like T cells that preserve the signatures of stem cell-like memory cells in the tumour environment [51,52]. To test if Th17 cells could be transformed into T_SM_ cells during BCG vaccination, we examined the expression of IL-17 in T_SM_ cells. We gated cells on CD45RA^+^CD62L^+^ naïve phenotype cells and analysed IL-17 production among CD95^+^ cells. We found that animals receiving BCG revaccination and clofazimine contained significantly higher numbers of IL-17-producing T_SM_ cells compared with all other groups (**Fig 6A**). Furthermore, to provide further evidence for the notion that IL-17-producing cells are Th17 cells and gradually differentiated into T_SM_ cells, we investigated the expression of RORγt, the signature transcription factor of Th17 cells, and *tcf-7*, a downstream component of the Wnt/β-catenin pathway that promotes cell plasticity. We found that clofazimine treatment during BCG revaccination upregulated both RORγt and *tcf-7* expression (**Fig 6B**). This result is in agreement with a published report that in the cancer microenvironment, IL-17-producing Th17 cells transform into T_SM_ cells due to expression of *tcf-7* [47]. We also analysed expression of *t-bet*, which is the signature marker of Th1 cells, to test the possibility that Th17 cells that transform into T_SM_ cells may eventually convert into host-protective Th1 cells. Surprisingly, we found significantly increased *tcf-7* and *t-bet* expression in T cells from mice that received BCG revaccination and clofazimine (**Fig 6B**). Recent studies have also shown that plasticity of Th17 cells has a role in acquiring enhanced memory T cell responses [51,53]. To confirm our result, we determined the status of another marker for cell plasticity, CD27. We found that clofazimine treatment significantly enhanced the level of CD27 expression in IL-17-producing cells (**Fig 6C)**. To provide further support for this notion, and to provide insight into the capacity of IL-17-producing cells to transform into IFN-γ-producing cells, we investigated the status of IL-17 expression among IFN-γ-producing cells. Our data clearly showed a 3-fold enhancement in the expression of IL-17^+^IFN-γ^+^ dual-positive cells in clofazimine-treated BCG***-***revaccinated animals compared with BCG-immunized or BCG-revaccinated animals (**Fig 6D**). Therefore, these findings suggest that clofazimine treatment during BCG revaccination enhances IL-17-producing T cells, which are then converted to T_SM_ cells that provide long-term memory responses by converting into host-protective, IFN-γ-producing Th1 cells.

## Discussion

*M*.*tb* has co-evolved with its human host since the pre-historic era [54] and has acquired various mechanisms to alter host protective immune responses so as to survive and replicate within the host. BCG, the only usable vaccine available, has been used since its inception in 1921, but its efficacy against adult pulmonary TB is unsatisfactory. However, it is sufficiently efficacious to protect against tubercular meningitis and disseminated TB in young children. The lack of BCG’s efficacy against adult pulmonary TB might be due to: (i) insufficient strength of the immune response, (ii) short-lived immune response, (iii) induction of anergy in memory T cells due to high antigen exposure, and/or (iv) antigen-specific memory cells are present in the periphery but fail to migrate to the site of infection in the lung. Previously, we and others have shown that BCG mounts strong Th1 responses but mounts only limited Th17 responses, which are required for optimal vaccine efficacy [17,18].

In most areas of the world where TB is prevalent vaccination with BCG is required by law. Although BCG induces a small pool of long-term T_CM_ cells, these cells become exhausted over time due to chronic exposure by environmental mycobacteria [17–19]. Recently, we showed that clofazimine increased the T_CM_ pool in BCG-vaccinated animals [28]. A recent clinical trial further showed that revaccination of BCG during adolescence increases TB protective immune responses over a single neonatal dose of BCG vaccination in a high transmission setting [55]. However, considerable literature suggests that addition of an immunomodulator improves BCG vaccine efficacy [14]. Based on these concepts we treated mice with a low dose of clofazimine during BCG revaccination, which similarly caused an increase in the T_CM_ population without significant alterations in the T_EM_ population. Recently, the quality of memory T cell responses has been evaluated by measuring antigen-specific cytokine production. Although cytokine production is an important function of memory T cells, their homing capacity is critical as well. Therefore, we investigated the polyfunctional properties of T_CM_ and T_EM_ cells. Prior studies have established that TNF-α^+^IFN-γ^+^IL-2^+^ triple-positive, polyfunctional T cells exhibit the most potent protection against TB [45]. In this context, some reports indicated that TNF-α^+^IFN-γ^+^ double-positive T cells correlate with high bacterial burden, whereas IFN-γ^+^IL-2^+^ or IL-2^+^ T cells correlate with low bacterial burden and increased long-term memory responses [56]. Our results demonstrated that clofazimine treatment enhanced IFN-γ^+^TNF-α^+^IL-2^+^ triple-positive polyfunctional T cells within both the T_CM_ and T_EM_ cell compartments, which give rise to long-term memory responses. Moreover, these cells did not exhibit an exhausted phenotype and were functionally active.

Recently, another subset of memory T cells was found which possesses characteristics of both naïve T cells and stem cells, called T_SM_ cells. This subset of memory T cells was identified for CD8^+^ T cells during virus infection [20,22]. Further studies also identified this subset in the CD4^+^ T cell population [20,22,29]. The most exciting character of T_SM_ cells is that they can differentiate into T_CM_ or T_EM_ cells and can proliferate extensively. T_SM_ cells are the only memory T cell subset with the ability to reconstitute the entire heterogeneous population of memory T cells [22,57]. Thus, T_SM_ cells are now considered the most important T cell subset for providing long-term memory [22]. Our study showed that this memory T cell subset is expanded by clofazimine following BCG revaccination. Interestingly, these T_SM_ cells preserved the molecular signature of Th17 cells, which likely contributes to disease protection [50,58]. Previous reports have shown that Th17 cells exhibit activated Wnt/β-catenin signalling that helps in restricting T cells to the naïve compartment and promotes the generation of T_SM_ cells [47,51]. It has also been shown that the molecular signature of Th17 cells can be down regulated with concomitant upregulation of Th1 type transcripts (i.e. tbx21 and IFN-γ) and ultimately such long-lived, ex-Th17 cells acquired T-bet expression and converted to Th1 cells [47]. In our study we found that clofazimine treatment enhances the frequency of T_SM_ cells as well as Th17 cells. It is now increasingly clear that Th17 cells transform into long-lived and less differentiated memory T cells, which in the long-term behave very similar to memory cells that differentiated from Th1 cells. Despite having limited plasticity Th17 cells mount superior recall responses as compared with Th1 cells. Due to activation of Wnt/β-catenin signalling and Tcf-7 expression in Th17 cells these cells exhibit enhanced plasticity [47] and might de-differentiate into naïve-like or early memory T cells. Here, we provided evidence that clofazimine enhances the plasticity of Th17 cells during BCG revaccination. This strategy maintains a stable pool of T_CM_ or T_EM_ cells due to increases in the T_SM_ population. Our previous work along with that of other groups has shown that Th1 and Th17 cell responses are predominantly required for enhanced protective memory T cell responses [12]. Improved memory responses were generated by suppressing Th2 and Treg cells [14]. In this study we found a trend for increased Th1 and Th17 cells and reduced Treg and Th2 cells induced by clofazimine after BCG revaccination. Cytokine analysis from culture supernatant also confirmed that host-protective Th1 and Th17 cells were increased in animals that received BCG revaccination and clofazimine, as levels of the Th1 type cytokines IL-12 and IFN-γ and the Th17 polarizing cytokines IL-1β and IL-6 were increased [59]. In this context, several prior recent studies have demonstrated that the combination of TGF-β and IL-6 promotes the differentiation of Th17 cells and inhibits Treg cell differentiation in mice [48,59], whereas TGF-β plus retinoic acid inhibits Th17 cell differentiation and promotes Treg cells [60]. The enhanced levels of IL-6, IL-1β and moderate levels of TGF-β found in mice treated with clofazimine and BCG revaccination might aid in the differentiation of Th17 cells with a reciprocal decrease in Treg cell differentiation. This pattern of Th cell subsets might induce superior memory T cell responses that provide long-term protection against *M*.*tb* infection. Furthermore, increased Th17 cells may help in the generation of T_SM_ cells, as found in other disease models [50]. It is also apparent that T_SM_ cells convert into T_CM_ or T_EM_ cells as well as produce long-term memory responses by rejuvenating themselves. Although it appears that T_SM_ cells are critical for long term vaccine efficacy, harnessing these cells for vaccine improvement is yet to be accomplished.

In summary, since optimal host protection against TB requires Th1 and Th17 responses, vaccine candidates should aim at inducing T_CM_ cells capable of producing Th1 and Th17 cytokines. Since T_EM_ cells give rise to effector cells that may cause inflammation, vaccines that promote T_CM_ over T_EM_ responses might be desired. Recent reports have indicated that T_CM_ and T_EM_ cells are derived from T_SM_ cells, a subset of memory long-lived, self-renewing memory T cells. Therefore, a successful vaccine candidate might involve T_SM_ cells that can give rise to both Th1 and Th17 cytokine-producing T cells. Since testing new vaccine candidates takes several decades, approaches that employ individuals previously vaccinated with BCG are meritorious. Our novel approach employs clofazimine, an approved drug, which showed efficacy for enhancing protective immune responses during revaccination in mice. A similar approach could be employed to enhance BCG vaccine efficacy in humans and warrants further investigation.

## Supporting information

S1 Fig Residual clofazimine *of serum* does not interfere with bacterial clearance. To determine if residual clofazimine is left in our experimental system, we immunized animals with BCG and rested for 60 days, as the similar manner of vaccine efficacy experiment. Similarly, a single BCG booster dose was given followed by clofazimine treatment (1 injection/week) for one month at a dose of 5 mg/kg. These animals were rested for additional 30 days. After 5 days and 30 days from the end of the treatment period we sacrifized animals and quantified the residual clofazimine in the blood serum by spectroscopy. As a negative control serum of uninfected and untreated mice were used and as a positive control serum from uninfected mice were collected after 5 days from one month dose (5 mg/kg) of clofazimine treatment for spectroscopic analysis.
(TIF)

S2 Fig Residual levels of clofazimine in fat tissues of clofazimine-treated animals. To determine the residual clofazimine in fat bodies we employed four groups of animals, with two groups that received an intraperitoneal clofazimine treatment for one month (1 injection/week) at a dose of 5 mg/kg, and two other groups that received vehicle control. After 5 days and 25 days from the end of the treatment period we sacrifized clofazimine-treated animals and quantified the residual clofazimine in fat bodies by spectroscopy. As negative control fat bodies from vehicle control animals were used for spectroscopic analysis. (a) Photographs of fat tissues from animals used for experiments after 5 days of treatment. (b) Spectroscopic analysis of residual clofazimine after 5 days of treatment. (c) Photographs of fat tissues from animals used for experiments after 25 days of treatment. (d) Spectroscopic analysis of residual clofazimine after 25 days of treatment. Red and blue lines in spectroscopic analyses are technical replicates of the sample.
(TIF)

S3 Fig Clofazimine has substantial immune modulatory effects in in vitro cultures. Spleens were harvested from H37Rv infected animals after 30 days and were challenged with M.tb lysate (CSA) in the presence of either INH or clofazimine, and stained with anti-CD4, -CD44, -CD62L, -IFN-γ and anti- IL-17 antibodies and analysed by FACS. (a) Schematic representation of FACS data and bar graphs for percentage of CD4^+^CD44^hi^CD62L^hi^ T_CM_ cells. (b) Schematic representation of FACS data and bar graphs for percentage of IL-17-producing CD4^+^ T cells. (c) Schematic representation of FACS data and bar graphs for percentage of IFN-γ-producing CD4^+^ T cells. All values are represented as mean±SD. Statistical analyses were performed by ANOVA with Tukey’s post hoc test. * denotes P ≤0.05. INH, Isoniazid.
(TIF)

S4 Fig Clofazimine enhances the expression of IL-7 cytokine receptor-α CD127 in BCG revaccinated animals. Spleens were harvested at different time points after aerosol challenge. Single cell suspensions were made. Cells were cultured overnight with *M*.*tb* lysate (CSA), and stained with anti-CD3, anti-CD4, -CD8, -CD44 and -CD62L antibodies for analysis of T_CM_ and T_EM_ by FACS. After obtaining T_CM_ cells we measured IL-7 cytokine receptor α (CD127) expression. All data are representative of 3 independent experiments and each group included at least 5 mice in each experiment. All values are represented as Mean±SD. Statistical analyses were performed by ANOVA with Tukey’s post hoc test. In this figure comparisons of CD127 expression in CD44^hi^CD62L^hi^ CD4^+^ or CD8^+^ lineage T cells were done between the BCG+BCG revaccination+CF+H37Rv group and all other experimental groups. * denotes P ≤0.05. CF, clofazimine.
(TIF)

S5 Fig Clofazimine enhances the T_CM_ population in the lungs of BCG re-immunized mice. At 60 days post infection lungs were harvested and single cell suspensions were made. Cells were cultured overnight with *M*.*tb* lysate (CSA). Cells were stained with anti-CD4, -CD8, -CD62L, and -CD44 antibodies and analysed by flow cytometry. Percentage of CD44^hi^CD62^lo^ T_EM_ and CD44^hi^CD62L^hi^ T_CM_ cells among CD4^+^ (a) and CD8^+^ (b) T cells. All data are representative of 3 independent experiments and each group included at least 5 mice in each experiment. All values are represented as Mean±SD. Statistical analyses were performed by ANOVA with Tukey’s post hoc test. In this figure comparisons of T_CM_ cells of CD4^+^ or CD8^+^ lineage cells were done between the BCG+BCG revaccination+CF+H37Rv group and all other experimental groups. * denotes P ≤0.05. CF, clofazimine.
(TIF)

S6 Fig Antigen-specific activated CD4^+^ cells in clofazimine-treated and BCG-vaccinated animals do not have an exhausted phenotype. Spleens were harvested and single cell suspensions were made. Cells were cultured overnight with *M*.*tb* lysate (CSA) and stained with anti-CD4, -PD1 or -Tim3 antibodies. Expression of PD1 and Tim3 was determined by FACS analysis. All data are representative of 3 independent experiments and each group included at least 5 mice in each experiment. All values are represented as Mean±SD. Statistical analyses were performed by ANOVA with Tukey’s post hoc test. In this figure comparisons of inhibitory molecules PD1 and Tim3 expression in CD4^+^ lineage cells were done between the BCG+BCG revaccination+CF+H37Rv group and all other experimental groups. *denotes P ≤0.05. CF, clofazimine.
(TIF)

S7 Fig Unstained controls of different experiments. (i) Unstained control of T_CM_ and T_EM_ analysis of (a) CD4 and (b) CD8 gated cells. (ii. a) Unstained control of CD95 and CD122 gated T_SM_ cells, (ii. b) unstained control of IL-6- and TGF-β***-***producing peritoneal macrophage cells and (ii. c) IL-17-producing CD4^+^ cells. (iii. a) Unstained control of IFN-γ***-*** or IL-4-producing CD4^+^ cells and (iii. b) IL-17-producing CD4^+^ cells. (iii. c) Unstained control of CD4^+^CD25^+^ cells and FOXP3-expressing cells in CD4^+^CD25^+^ cells. (iv. a) Unstained control of IL-17-producing cells with signature marker CD95 of T_SM_ cells. (iv. b) Unstained control of IL-17-producing cells expressing CD27 in CD4^+^ cells and (iv. c) unstained control of IL-17 and IFN-γ dual cytokine-producing CD4^+^ cells.
(TIF)
