# Supplementary figures and images for: Clofazimine enhances the efficacy of BCG revaccination via stem cell-like memory T cells
Source: PLoS Pathog. 2020 May 21;16(5):e1008356. doi: 10.1371/journal.ppat.1008356 (PMC7269335; doi:10.1371/journal.ppat.1008356)

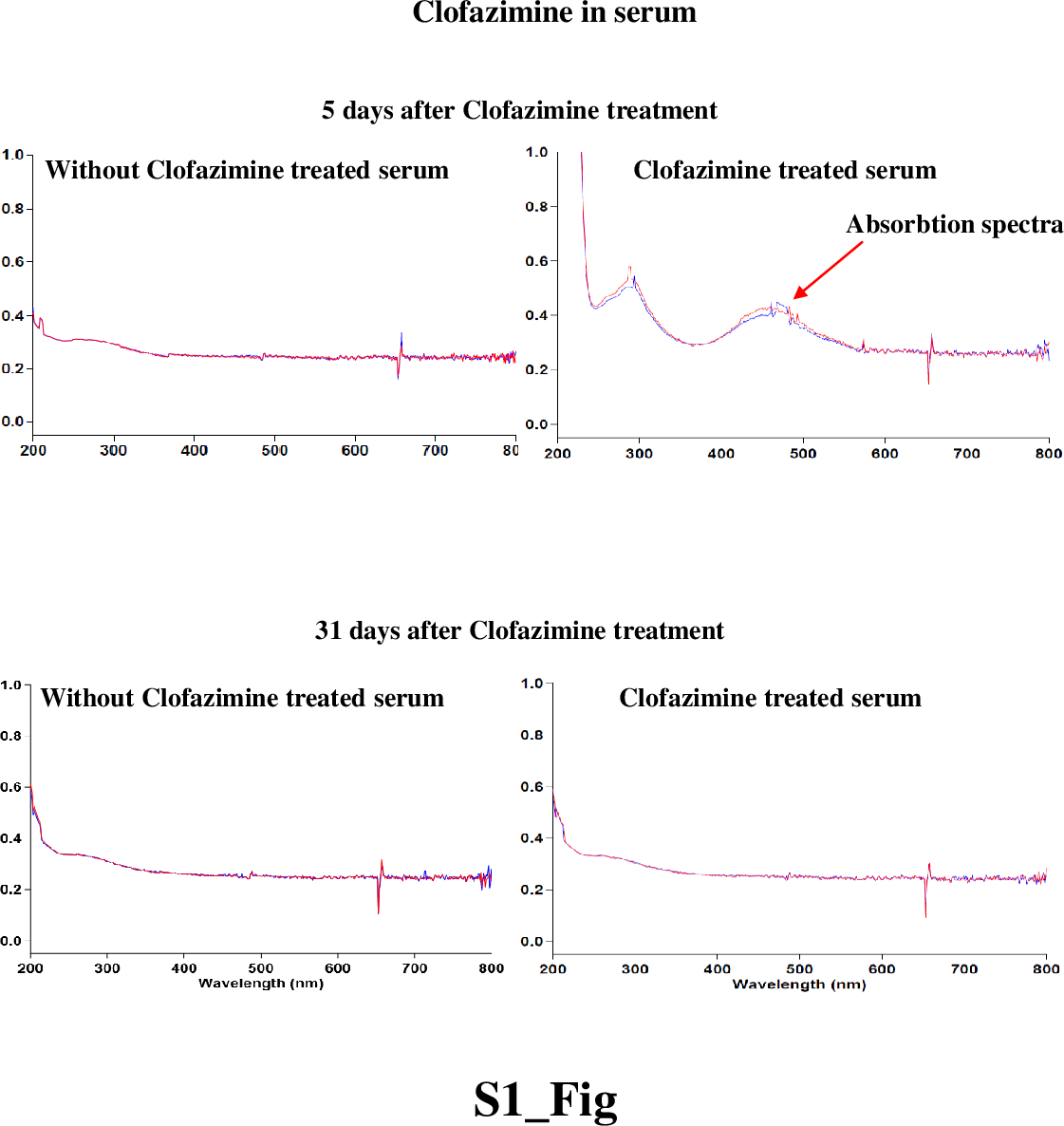

Supplement: S1 Fig — To determine if residual clofazimine is left in our experimental system, we immunized animals with BCG and rested for 60 days, as the similar manner of vaccine efficacy experiment. Similarly, a single BCG booster dose was given followed by clofazimine treatment (1 injection/week) for one month at a dose of 5 mg/kg. These animals were rested for additional 30 days. After 5 days and 30 days from the end of the treatment period we sacrifized animals and quantified the residual clofazimine in the blood serum by spectroscopy. As a negative control serum of uninfected and untreated mice were used and as a positive control serum from uninfected mice were collected after 5 days from one month dose (5 mg/kg) of clofazimine treatment for spectroscopic analysis. (TIF) [file ppat.1008356.s001.tif]

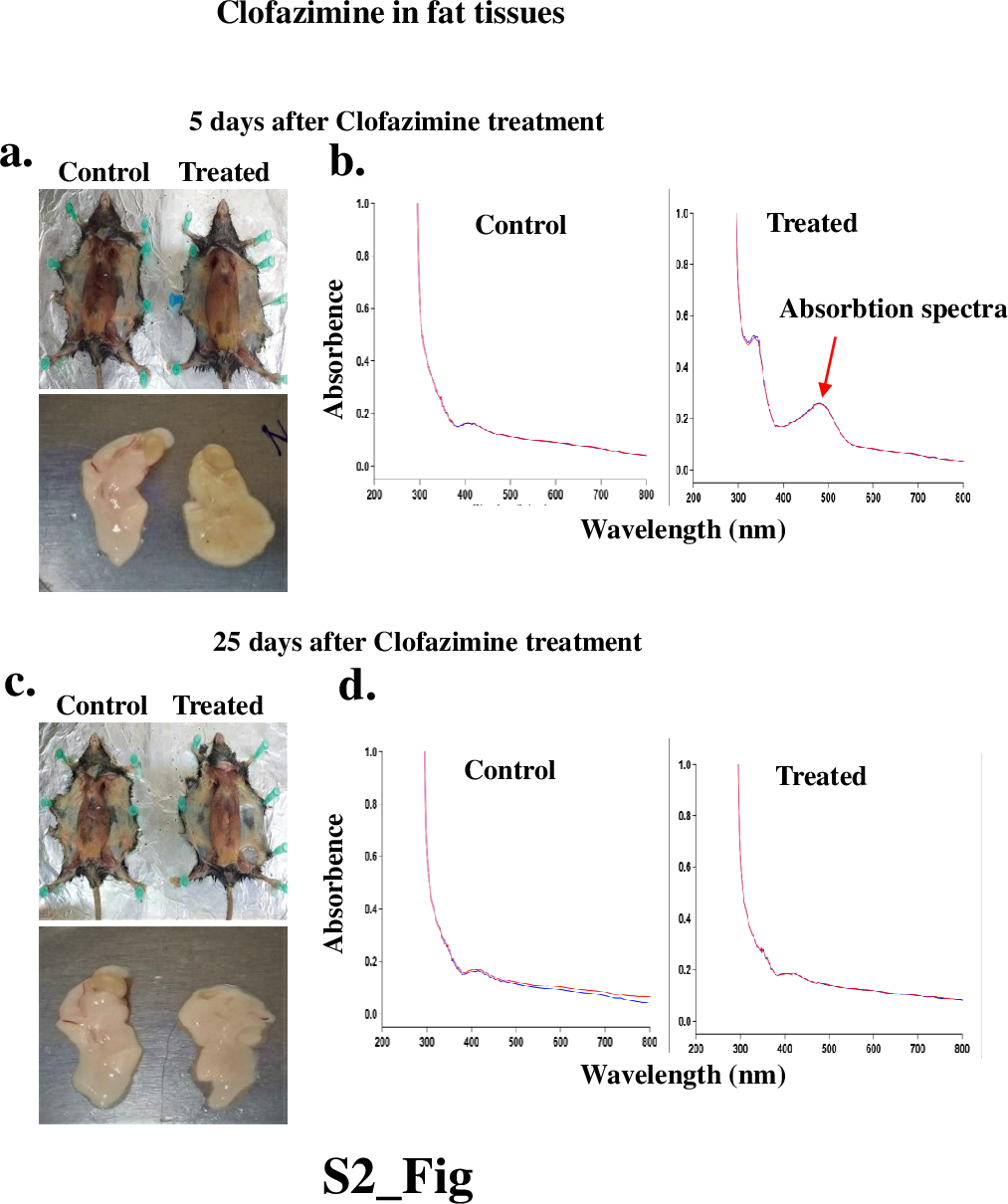

Supplement: S2 Fig — To determine the residual clofazimine in fat bodies we employed four groups of animals, with two groups that received an intraperitoneal clofazimine treatment for one month (1 injection/week) at a dose of 5 mg/kg, and two other groups that received vehicle control. After 5 days and 25 days from the end of the treatment period we sacrifized clofazimine-treated animals and quantified the residual clofazimine in fat bodies by spectroscopy. As negative control fat bodies from vehicle control animals were used for spectroscopic analysis. (a) Photographs of fat tissues from animals used for experiments after 5 days of treatment. (b) Spectroscopic analysis of residual clofazimine after 5 days of treatment. (c) Photographs of fat tissues from animals used for experiments after 25 days of treatment. (d) Spectroscopic analysis of residual clofazimine after 25 days of treatment. Red and blue lines in spectroscopic analyses are technical replicates of the sample. (TIF) [file ppat.1008356.s002.tif]

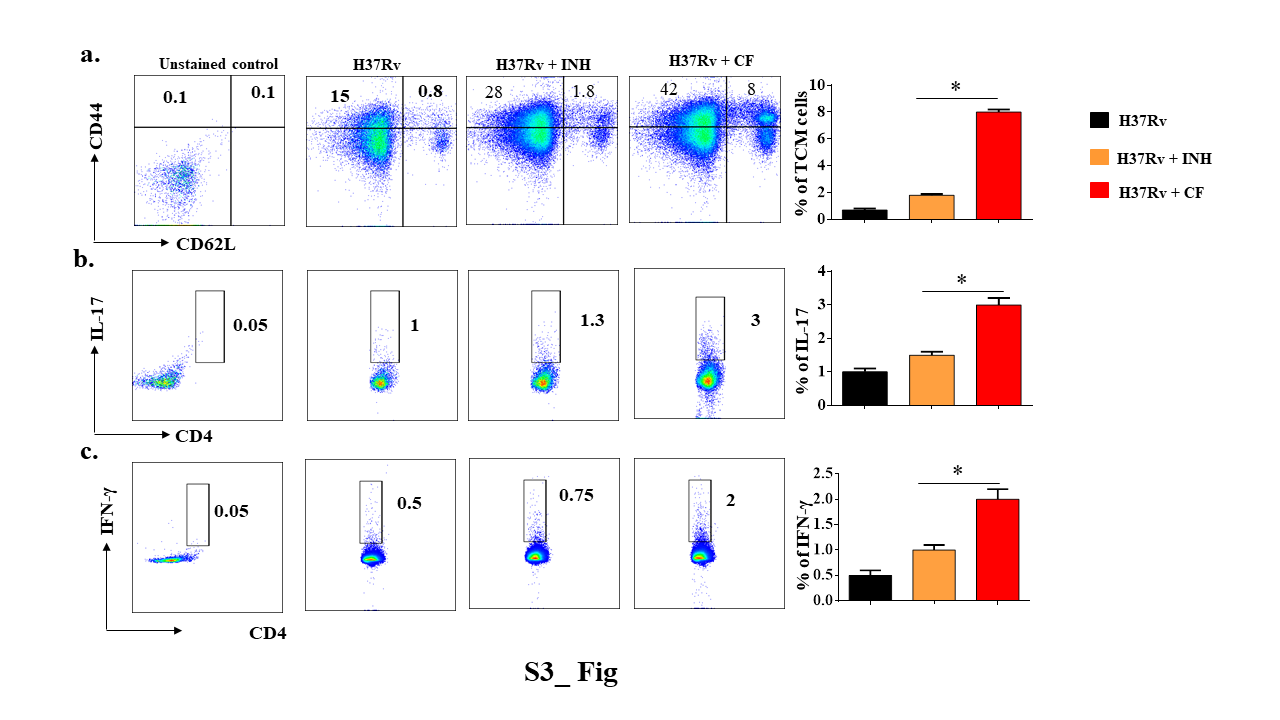

Supplement: S3 Fig — Spleens were harvested from H37Rv infected animals after 30 days and were challenged with M.tb lysate (CSA) in the presence of either INH or clofazimine, and stained with anti-CD4, -CD44, -CD62L, -IFN-γ and anti- IL-17 antibodies and analysed by FACS. (a) Schematic representation of FACS data and bar graphs for percentage of CD4+CD44hiCD62Lhi TCM cells. (b) Schematic representation of FACS data and bar graphs for percentage of IL-17-producing CD4+ T cells. (c) Schematic representation of FACS data and bar graphs for percentage of IFN-γ-producing CD4+ T cells. All values are represented as mean±SD. Statistical analyses were performed by ANOVA with Tukey’s post hoc test. * denotes P ≤0.05. INH, Isoniazid. (TIF) [file ppat.1008356.s003.tif]

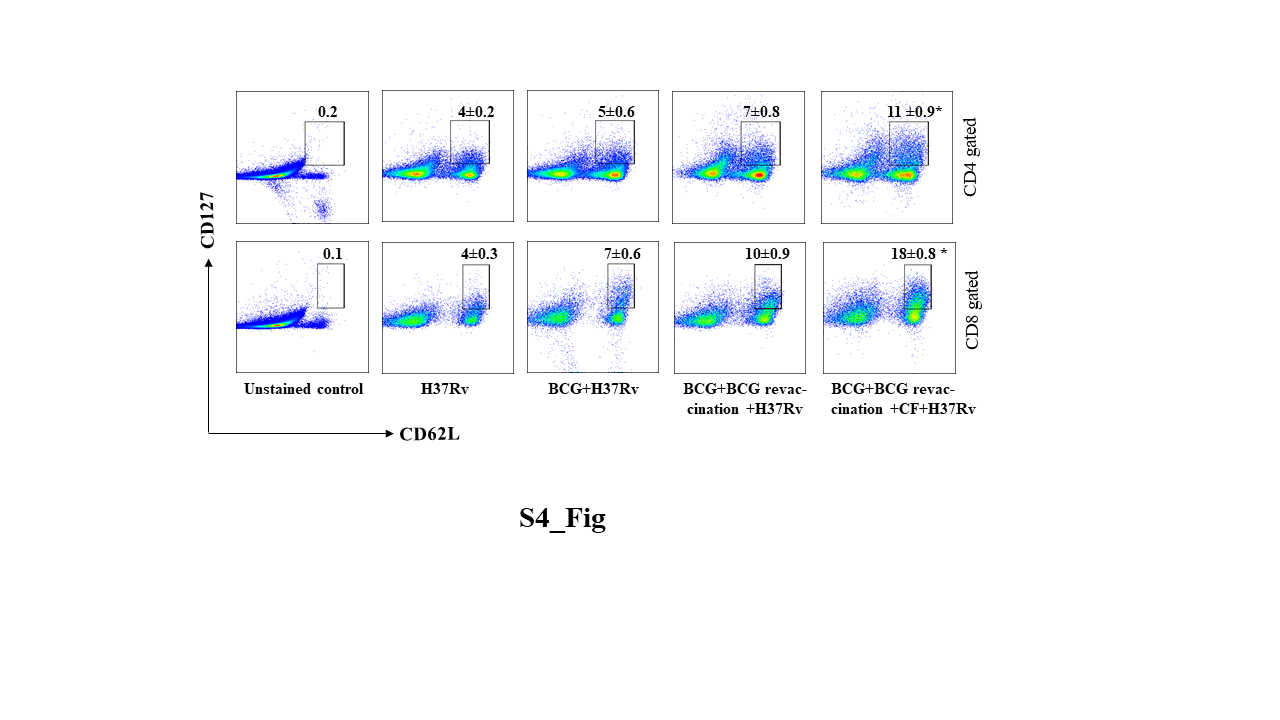

Supplement: S4 Fig — Spleens were harvested at different time points after aerosol challenge. Single cell suspensions were made. Cells were cultured overnight with M.tb lysate (CSA), and stained with anti-CD3, anti-CD4, -CD8, -CD44 and -CD62L antibodies for analysis of TCM and TEM by FACS. After obtaining TCM cells we measured IL-7 cytokine receptor α (CD127) expression. All data are representative of 3 independent experiments and each group included at least 5 mice in each experiment. All values are represented as Mean±SD. Statistical analyses were performed by ANOVA with Tukey’s post hoc test. In this figure comparisons of CD127 expression in CD44hiCD62Lhi CD4+ or CD8+ lineage T cells were done between the BCG+BCG revaccination+CF+H37Rv group and all other experimental groups. * denotes P ≤0.05. CF, clofazimine. (TIF) [file ppat.1008356.s004.tif]

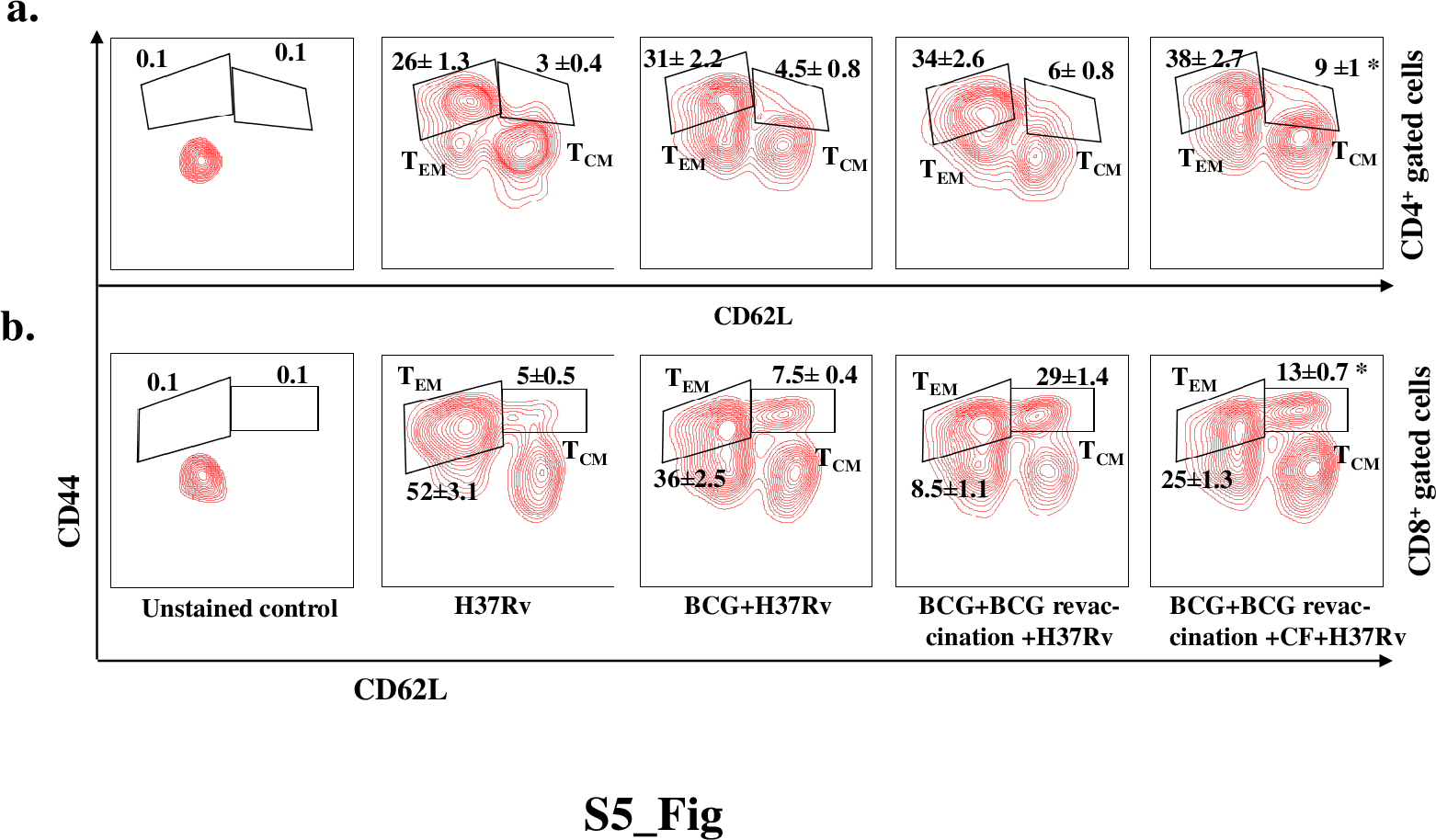

Supplement: S5 Fig — At 60 days post infection lungs were harvested and single cell suspensions were made. Cells were cultured overnight with M.tb lysate (CSA). Cells were stained with anti-CD4, -CD8, -CD62L, and -CD44 antibodies and analysed by flow cytometry. Percentage of CD44hiCD62lo TEM and CD44hiCD62Lhi TCM cells among CD4+ (a) and CD8+ (b) T cells. All data are representative of 3 independent experiments and each group included at least 5 mice in each experiment. All values are represented as Mean±SD. Statistical analyses were performed by ANOVA with Tukey’s post hoc test. In this figure comparisons of TCM cells of CD4+ or CD8+ lineage cells were done between the BCG+BCG revaccination+CF+H37Rv group and all other experimental groups. * denotes P ≤0.05. CF, clofazimine. (TIF) [file ppat.1008356.s005.tif]

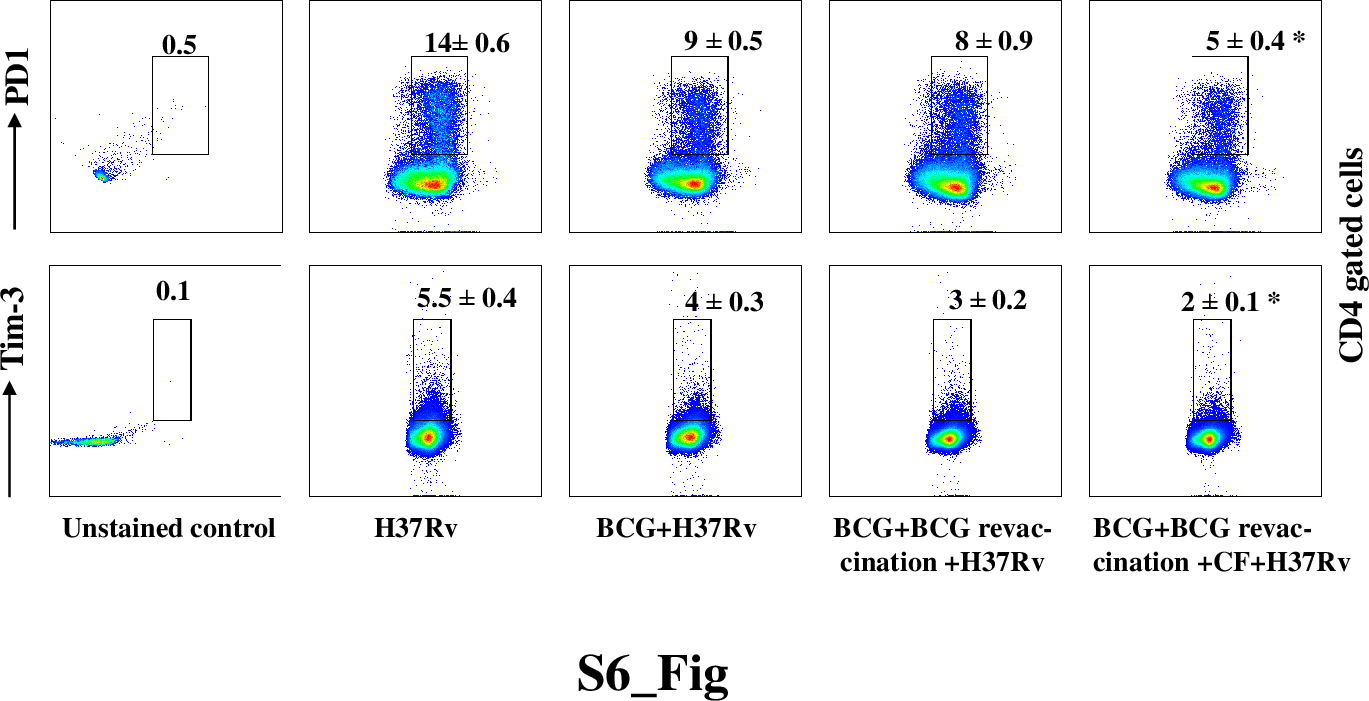

Supplement: S6 Fig — Spleens were harvested and single cell suspensions were made. Cells were cultured overnight with M.tb lysate (CSA) and stained with anti-CD4, -PD1 or -Tim3 antibodies. Expression of PD1 and Tim3 was determined by FACS analysis. All data are representative of 3 independent experiments and each group included at least 5 mice in each experiment. All values are represented as Mean±SD. Statistical analyses were performed by ANOVA with Tukey’s post hoc test. In this figure comparisons of inhibitory molecules PD1 and Tim3 expression in CD4+ lineage cells were done between the BCG+BCG revaccination+CF+H37Rv group and all other experimental groups. *denotes P ≤0.05. CF, clofazimine. (TIF) [file ppat.1008356.s006.tif]

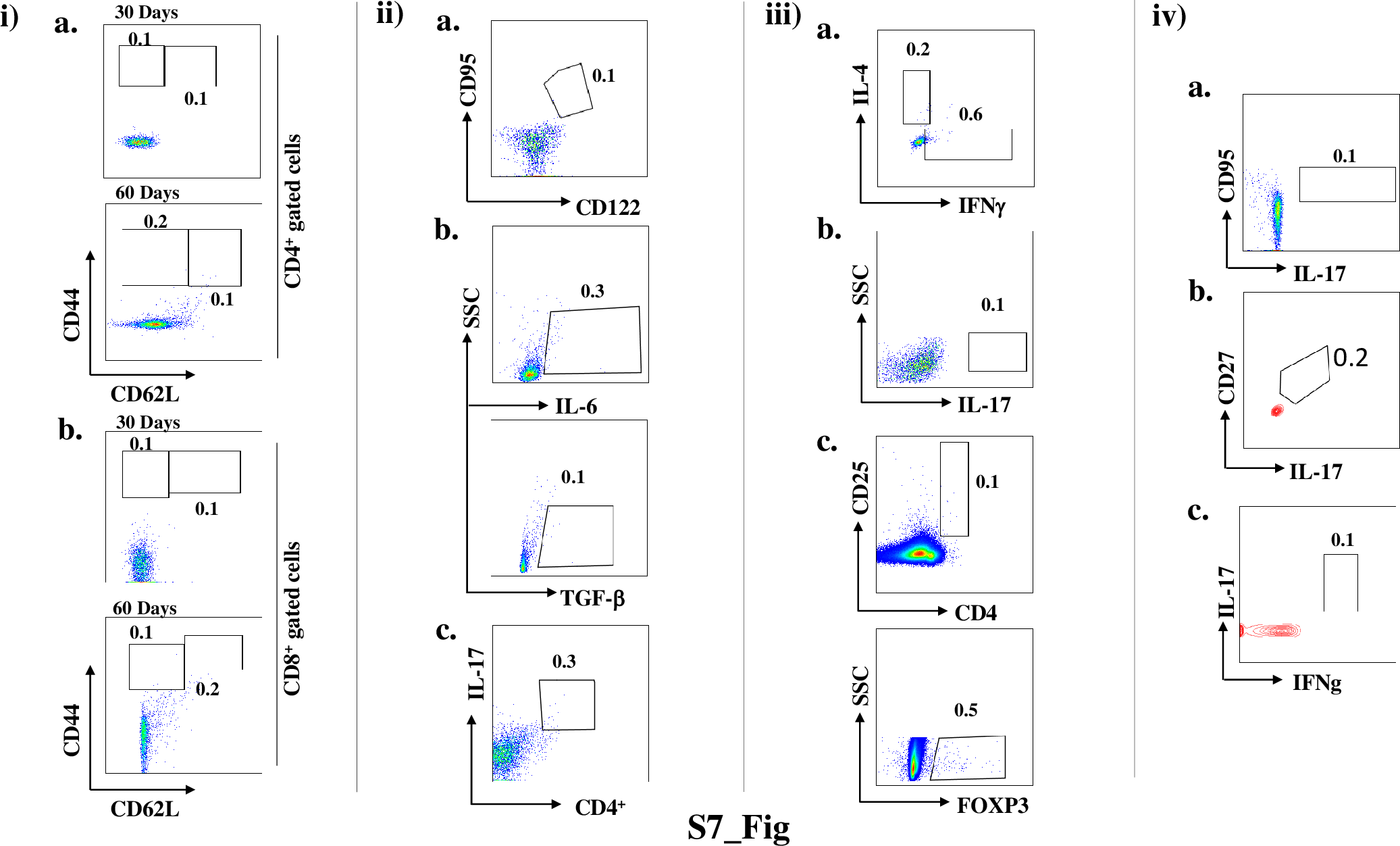

Supplement: S7 Fig — (i) Unstained control of TCM and TEM analysis of (a) CD4 and (b) CD8 gated cells. (ii. a) Unstained control of CD95 and CD122 gated TSM cells, (ii. b) unstained control of IL-6- and TGF-β-producing peritoneal macrophage cells and (ii. c) IL-17-producing CD4+ cells. (iii. a) Unstained control of IFN-γ- or IL-4-producing CD4+ cells and (iii. b) IL-17-producing CD4+ cells. (iii. c) Unstained control of CD4+CD25+ cells and FOXP3-expressing cells in CD4+CD25+ cells. (iv. a) Unstained control of IL-17-producing cells with signature marker CD95 of TSM cells. (iv. b) Unstained control of IL-17-producing cells expressing CD27 in CD4+ cells and (iv. c) unstained control of IL-17 and IFN-γ dual cytokine-producing CD4+ cells. (TIF) [file ppat.1008356.s007.tif]
